# AID-Targeting and Hypermutation of Non-Immunoglobulin Genes Does Not Correlate with Proximity to Immunoglobulin Genes in Germinal Center B Cells

**DOI:** 10.1371/journal.pone.0039601

**Published:** 2012-06-29

**Authors:** Hillary Selle Gramlich, Tara Reisbig, David G. Schatz

**Affiliations:** 1 Department of Cell Biology, Yale University School of Medicine, New Haven, Connecticut, United States of America; 2 Department of Immunobiology, Yale University School of Medicine, New Haven, Connecticut, United States of America; 3 Howard Hughes Medical Institute, Chevy Chase, Maryland, United States of America; University of Miami, United States of America

## Abstract

Upon activation, B cells divide, form a germinal center, and express the activation induced deaminase (AID), an enzyme that triggers somatic hypermutation of the variable regions of immunoglobulin (Ig) loci. Recent evidence indicates that at least 25% of expressed genes in germinal center B cells are mutated or deaminated by AID. One of the most deaminated genes, *c-Myc*, frequently appears as a translocation partner with the Ig heavy chain gene (*Igh*) in mouse plasmacytomas and human Burkitt's lymphomas. This indicates that the two genes or their double-strand break ends come into close proximity at a biologically relevant frequency. However, the proximity of *c-Myc* and *Igh* has never been measured in germinal center B cells, where many such translocations are thought to occur. We hypothesized that in germinal center B cells, not only is *c-Myc* near *Igh*, but other mutating non-Ig genes are deaminated by AID because they are near Ig genes, the primary targets of AID. We tested this “collateral damage” model using 3D-fluorescence *in situ* hybridization (3D-FISH) to measure the distance from non-Ig genes to Ig genes in germinal center B cells. We also made mice transgenic for human *MYC* and measured expression and mutation of the transgenes. We found that there is no correlation between proximity to Ig genes and levels of AID targeting or gene mutation, and that *c-Myc* was not closer to *Igh* than were other non-Ig genes. In addition, the human *MYC* transgenes did not accumulate mutations and were not deaminated by AID. We conclude that proximity to Ig loci is unlikely to be a major determinant of AID targeting or mutation of non-Ig genes, and that the *MYC* transgenes are either missing important regulatory elements that allow mutation or are unable to mutate because their new nuclear position is not conducive to AID deamination.

## Introduction

In mice and humans, antibodies are produced by B cells and are composed of two light chains, coded for by either *Igκ* or *Igλ*, and two heavy chains, coded for by *Igh*. Each of the heavy and light chains consist of a variable region, which binds antigen and varies from B cell to B cell, and a constant region, whose sequence does not change for a given antibody isotype. There are several different *Igh* constant regions, conferring different effector functions, and the B cell can undergo class switch recombination (CSR), a rearrangement of germline DNA, to switch from one constant region to another. The regions of DNA that break during CSR are called switch regions and, with the exception of IgD, every constant region has its own upstream switch region [1].

Both CSR and somatic hypermutation (SHM) are initiated by and dependent on the enzyme Activation Induced Cytidine Deaminase (AID). After a mature B cell is activated by pathogen recognition and T cell interaction, it divides in the spleen or lymph node and can contribute to the formation of a cluster of cells called a germinal center (GC). GC B cells express AID, which deaminates deoxycytidine in the variable region and switch region DNA, thereby generating deoxyuracil:deoxyguanine (dU:dG) mismatches. The dU:dG lesions can then be recognized by the base excision repair protein Uracil DNA Glycosylase (UNG) and the mismatch repair proteins Msh2/6 [2], [3].

Although UNG and Msh2/6 usually initiate high-fidelity lesion repair, during SHM of variable regions in GC B cells they trigger error-prone repair processes, thereby spreading and changing the nature of the AID induced lesions and yielding a mutated variable region [4]. During CSR, these same repair factors process the dU residues introduced by AID in switch regions to create DNA double-strand breaks, whose processing and rejoining leads to CSR [5]. SHM is also associated with double strand breaks [6], [7], [8]. Variable region hypermutation is of benefit to the organism since it allows the generation of B cells with slightly altered variable regions and antigen specificity. Some of the new B cells are likely to have a higher affinity for the invading pathogen and such B cells are enriched for during the GC response in a process known as affinity maturation.

AID is expressed at high levels only in GC B cells and targets immunoglobulin gene variable and switch regions at a level far higher than other genes, but AID mediated mutation is not limited to Ig genes [9]. SHM has been observed in GC or memory B cells in the human genes *BCL6*, *CD95*, *CD79A*, and *CD79B* [10], [11], [12], [13], and a recent study in mice found that more than 20% of a large panel of expressed genes in WT GC B cells were mutated at a significant level [14]. Analysis of mutation in GC B cells from *Msh2^−/−^Ung^−/−^* double knockout mice, which show the footprint of AID action since lesions can neither be repaired nor spread to surrounding DNA, indicated that more than 50% of the expressed genes analyzed were targeted by AID [14]. Since this study found many genes whose mutation frequency was significantly higher in *Msh2^−/−^Ung^−/−^* GC B cells than in WT GC B cells, the authors proposed that there are three groups of genes: those not detectably deaminated by AID, those deaminated by AID but repaired predominantly by high-fidelity processes, and those deaminated by AID and further mutated by error-prone repair pathways [9], [14].

Despite the abundance of mutation in non-Ig genes, even the most highly mutated non-Ig gene, *Bcl6*, mutates at a frequency 25 times lower than that of *Igh* [14]. AID action is largely targeted to Ig variable and switch regions and the mechanism of this targeting is not yet well understood. The Ig variable region sequence and promoter are not critical for targeting [15], [16], [17], and a role for Ig enhancers is uncertain since their effect is only obvious in transgenes [18], [19], [20], [21], [22], [23], [24]. E2A proteins might be involved in AID targeting since E-box motifs, the binding sites for E2A proteins, accidentally incorporated into a transgene increased the mutation of that transgene [25]. Finally, there might be some role for histone modifications. Histone H4 acetylation and histone 3 lysine 9 trimethylation (H3K9me3) have been associated with switch regions undergoing CSR and H3K9me3 might be involved in tethering of AID to donor switch regions [26], [27], [28], [29]. No direct link has been established between a histone modification and AID recruitment to variable regions, but phosphorylation of serine 14 on histone 2B closely correlates with SHM and CSR, being elevated in the variable and switch regions of actively hypermutating GC B cells [30].

*c-Myc*, one of the genes found to be highly deaminated by AID in mouse GC B cells, is a common translocation partner with Ig genes in human and mouse. *c-Myc-Igh* translocations deregulate *c-Myc*, thereby contributing to mouse plasmacytomas and human Burkitt's lymphomas [31], [32], [33], [34], [35], [36]. It has been known for some time that the Ig gene translocation break points are in regions deaminated by AID; in humans, near Ig light chain variable regions or an *Igh* switch region, and in mice, near an *Igh* switch region [34], [35], [37], [38], [39], [40], [41], [42], [43], [44], [45], [46], [47], [48]. Interestingly, two studies found that the region of *c-Myc* deaminated by AID is precisely the region of *c-Myc* breakpoints in plasmacytomas and sporadic Burkitt's lymphomas - the first exon and first intron [14], [49].

Not only are the *c-Myc* and Ig breaks in regions deaminated by AID, but the translocations, and very likely the double strand breaks that underlie them, are also strongly dependent on AID [50], [51], [52]. Since translocations require both breakage and proximity, one might predict that *c-Myc* and *Igh* are near one another in the nucleus of GC B cells. In addition, another gene in the Myc family, *n-Myc*, does not translocate with *Igh* normally, but does so when moved to the *c-Myc* locus [53]. We expected that the *c-Myc* locus is conducive to *Igh* translocations at least in part because it is physically close to the *Igh* locus in cells expressing AID. *c-Myc* and *Igh* have been shown to move together for a short period of 5–10 minutes after ex-vivo stimulation of B cells [54], well before these cells express AID, but there have not been any studies assessing the proximity of the two genes in GC B cells, at the time of physiological, high-level AID expression.

We further hypothesized that proximity to Ig genes, in addition to influencing translocation frequency, plays a role in determining the mutation frequency of non-Ig genes. Specifically, we considered a “collateral damage” model in which Ig genes are targeted for high-level deamination and mutation, with the deamination and mutation of non-Ig genes depending in part on their distance to a mutating Ig gene. Indeed, it is known that a high level of AID is present at *Igh* switch regions during CSR [55], and the same is presumably true for other active Ig genes in GC B cells. A high concentration of AID and other factors necessary for mutation at Ig loci could increase the likelihood of mutation of nearby genes.

To test the role of relative nuclear position in SHM targeting, we used 3D-fluorescence in situ hybridization (3D-FISH) to determine the position of four genes deaminated by AID (*Bcl6*, *Cd83*, *Pim1*, and *c-Myc*) and two non-mutated/non-deaminated genes (*β2* *m* and *Mef2b*) relative to *Igh* and *Igλ* in splenic murine GC B cells. Our analysis also allowed us to test the model that *Igh* and *c-Myc* are prone to translocate with one another because they are in unusually close proximity in GC B cells. Further, we generated two mouse lines transgenic for a bacterial artificial chromosome (BAC) spanning the human *MYC* locus, bred the transgenes onto the *Msh2^−/−^Ung^−/−^* background, and assessed whether *MYC* was deaminated by AID and in close proximity to *Igh* in two new nuclear locations. Our results fail to reveal any correlation between the propensity of a gene to undergo SHM or deamination by AID and its proximity to a mutating Ig locus in GC B cells. The mouse *c-Myc* locus was not found to reside closer to *Igh* than the other loci analyzed, and the human *MYC* transgenes, though expressed in most cells analyzed, did not acquire mutations on either the WT or *Msh2^−/−^Ung^−/−^* background. Our results do not support the collateral damage model and indicate that stable and close association of a gene with *Igh* or *Igλ* in GC B cells is not necessary for mutation or targeting by AID.

## Results

### Distance between Non-Ig Loci and *Igh*

We measured the distance between non-Ig genes of interest and Ig genes in GC B cells by 3D-FISH using GC B cells isolated from spleens of B1-8 heterozygous mice immunized 10 days prior with the hapten NP conjugated to chicken gamma globulin (NP-CGG). B1-8 mice carry a knocked-in pre-rearranged *Igh* allele that is able to bind NP as long as a λ1 or λ3 light chain is expressed. The number of B cells that respond to NP is therefore substantially increased in B1-8 mice [56], [57], [58], [59]. After sorting for GC cells 8–16 days after NP immunization, we found that the greatest number of GC cells was obtained from the mice day 10 post-immunization (data not shown), and therefore used this time point for most experiments.

To enrich for GC B cells before purification by cell sorting, we negatively selected to remove non-B cells and some naïve B cells (see Materials and Methods). GC B cells were then sorted based on expression of B220, FAS, and binding to NIP, a hapten similar to NP which is bound by NP responding B cells. Naïve B cells, which were sorted from a spleen sample that had not been through the negative selection, were B220^+^ but FAS^lo^ and did not bind NIP. An example of a typical GC B cell purification is shown in Figure S1.

Mutation analysis showed that the pattern of mutation in spleen GC B cells is similar to that in GC B cells from Peyer's patches of 5–8 month old mice (Figure S2, Table S1). *Bcl6* is mutated more than are other non-Ig genes, followed by *Cd38* and *c-Myc*, all of which are above the background mutation frequency previously defined in AID-deficient GC B cells [14]. The mutation frequencies in GC B cells from Peyer's patch are higher than those in spleen, presumably because the former have been chronically stimulated by antigens in the gut.

**Figure 1:**
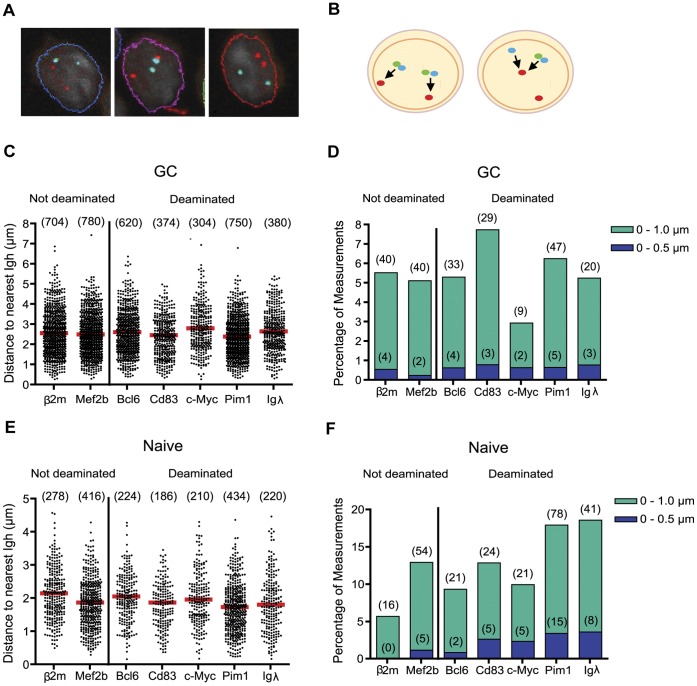
Distances of genes from *Igh* in germinal center and naïve B cells. (A) Examples of cells used in analysis. Collapsed z-stacks are shown with nuclei outlined. *Igh* probes are shown in red and *Bcl6* probes are shown in blue and green (colors overlap in example shown). In all experiments, only cells in which both alleles for both genes under study were clearly visible were included in the analysis. Ig alleles were marked by one probe and other genes were marked by either one or two probes. Cells whose nuclei were cut off by the edge of the picture were excluded. (B) Schematic showing measurements taken from cells. Two cells and their nuclei are shown with red dots representing FISH probes to an Ig gene and blue and/or green dots representing two different FISH probes to a non-Ig gene. Distances were measured from each allele of the gene of interest to the closest allele of the Ig gene in question. (C and E) Scatter plots showing all distances (µm) measured from the gene marked on the x-axis to the nearest *Igh* allele. The number of measurements taken is shown in parentheses above each data set and red bars mark the median. Two genes that are not deaminated by AID are shown on the left, and 5 genes that are deaminated by AID are shown on the right. (C) Measurements taken from splenic GC B cells 10 days post-immunization with 4-hydroxy-3-nitrophenylacetyl conjugated to chicken gamma globulin (NP-CGG). (E) Measurements taken from splenic naïve B cells collected from the same spleen samples as those used in (C). (D and F) Bar graphs summarizing data from (C) and (E) respectively. Bars represent the percentage of measurements in a dataset within 0.5 µm (dark blue) or 1.0 µm (blue green). The number of measurements found in each distance range are shown in parentheses.

We chose to use a 3D-FISH procedure that preserves the nuclear architecture and leaves nuclei spherical rather than flattened on the slide. This procedure also results in relatively small fluorescent spots marking each allele, thereby increasing the resolution of the analysis. We analyzed only cells in interphase, with the entire nucleus visible in the frame, and with both alleles of each gene marked by fluorescent probe (Figure 1A). In each experiment, we assessed one gene of interest and one Ig gene, and measured the distance from each allele of the gene of interest to the nearest allele of the Ig gene (Figure 1B).

We chose to analyze two genes that mutate in WT mice (*Bcl6* and *Cd83*), and two genes that are deaminated by AID but show much higher mutation frequencies in *Msh2^−/−^Ung^−/−^* double knockout mice than in WT mice (*c-Myc* and *Pim1*). In a large sequencing study, *Bcl6* and *Cd83* were the two most mutated non-Ig genes in WT mice, and *Bcl6*, *c-Myc*, *Pim1*, and *Cd83* were four of the five most deaminated genes in *Msh2^−/−^Ung^−/−^* double knockout mice [14]. All of the genes are expressed in GC B cells, though *c-Myc* is expressed only at low levels [60]. To determine whether mutating or deaminated genes are closer to Ig genes than are non-deaminated genes, we compared the distances measured from each of the four deaminated genes and an Ig gene to those measured from each of two non-deaminated genes (*β2* *m* and *Mef2b* [14]) and an Ig gene. We also measured the distance between *Igλ* and *Igh* to assess whether mutating Ig genes cluster in a single nuclear location.

The distance data for these six non-Ig genes relative to *Igh* in GC B cells are quite similar to one another (Figure 1C and 1D). Median distances are near 2.5 µm (Figure 1C, Table S2), and in all cases less than 1% of the gene-of-interest alleles were within 0.5 µm of an *Igh* allele (Figure 1D). These observations were also true of *Igλ-Igh* distance measurements (Figure 1C and 1D). There are some statistically significant differences between the datasets: *Cd83* and *Pim1* are slightly closer and *c-Myc* is slightly farther from *Igh* than is *β2* *m* (Table S3). However, no trend could be discerned to suggest that non-mutating genes are generally farther from *Igh* than are mutating genes.

We were primarily interested in the positions of the genes relative to Ig genes in GC B cells, but we also wondered how and if the distances might change in the transition from naïve to GC B cell. For example, it was conceivable that, prior to antigen activation, AID-deaminated genes were closer to Ig loci than were non-deaminated genes, thereby predisposing them to AID action. We therefore measured the distances from the genes of interest to the nearest *Igh* allele in naïve B cells and looked for differences between the deaminated and non-deaminated genes. Overall, the results in naïve B cells were similar to those in GC B cells. Because naïve B cells are smaller than GC B cells, all distances measured were on average smaller and the percentage of measurements within 1 µm were larger, but again AID deaminated genes were not consistently closer to *Igh* than were non-deaminated genes (Figure 1E and 1F, Tables S4 and S5).

**Figure 2:**
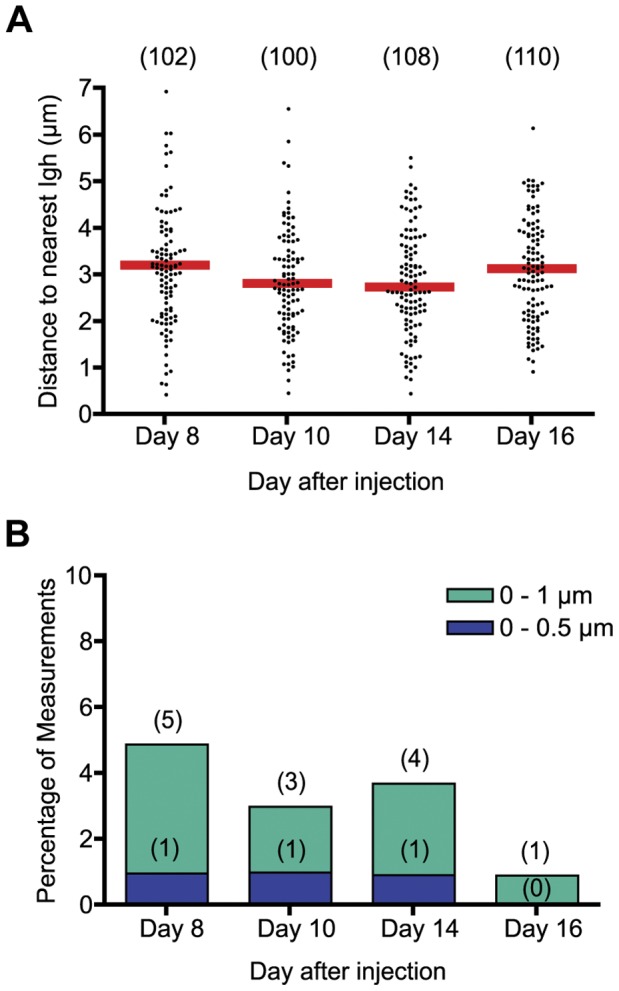
Distance between *c-Myc* and *Igh* during the GC response. (A) Scatter plot showing the distance (µm) between *c-Myc* and *Igh* in GC B cells 8, 10, 14, and 16 days post-immunization with NP-CGG. (B) Bar graphs summarizing data from (A). Data are presented as in Figure 1.

It is interesting that of all the non-Ig genes studied, the one farthest on average from *Igh* in GC B cells was *c-Myc*. Since many *c-Myc* and *Igh* translocations are AID mediated [3], [50], [51], [52], we thought it possible that at some point during the time of AID activation, *c-Myc* and *Igh* would tend to be near one another, and that perhaps analysis at day 10 of the GC response had failed to detect this. We therefore measured the distance between *c-Myc* and *Igh* at other times in the GC response, starting at day 8 post-immunization, which was the earliest that we could collect enough cells for analysis, and ending at day 16 (Figure 2). We collected data from fewer cells in the timecourse experiment than we had in the previous experiment, but we confirmed the validity of the data by collecting a new set of data at day 10 and comparing it to the previous day 10 data, yielding median distances between *c-Myc* and *Igh* of 2.78 and 2.75 µm, respectively, with a standard deviation of 1.14 µm for both datasets (compare Figure 1C with Figure 2A and Table S2 with Table S6). Our analysis of distance at day 8, 10, 14, and 16 post-immunization show that the median distance from *c-Myc* to *Igh* is not significantly shorter at any other time point than at day 10 (Figure 2A, Tables S6 and S7). It is therefore likely that *c-Myc* is at a greater distance on average from *Igh* throughout the GC response than are the other non-Ig genes assessed in this study. Furthermore, *c-Myc* and *Igh* are not within 0.5 µm of one another with any great frequency at any other time point examined than at day 10 (Figure 2B). There was a smaller percentage of measurements within 1.0 µm on day 10 relative to days 8 and 14, but since the datasets were small the actual number of measurements in range in each group was small, making differences on this level difficult to interpret.

To confirm that our methods were sufficiently sensitive to detect changes in locus proximity and to relate this to *c-Myc*, we replicated a previously published 3D-FISH experiment of Osborne et al. [54] in which the distance between *c-Myc* and *Igh* was examined in naïve B cells prior to and 5 minutes after ex vivo stimulation with IL-4, α-CD40, and α-IgM. In close agreement with their findings, we observed that the peak percentage of measurements shifted from the 1.6–2.4 µm range in unstimulated cells to the 0.8–1.6 µm range for 5 minute stimulated cells (data not shown).

### Distance between Non-Ig Loci and *Igλ*

**Figure 3:**
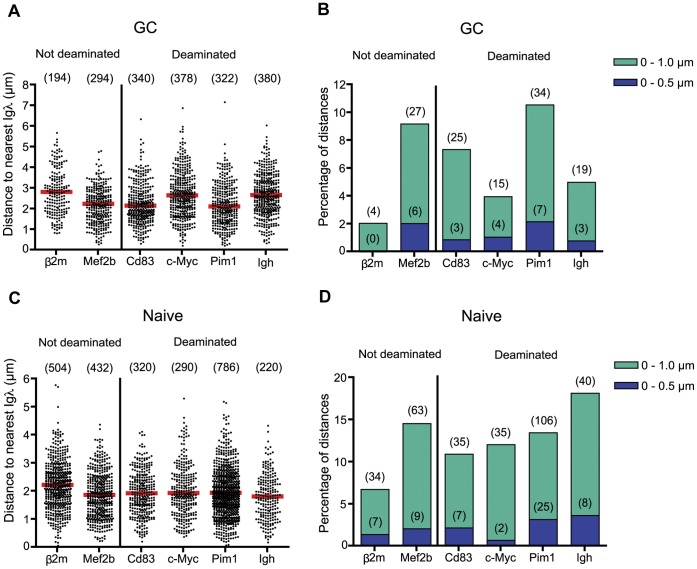
Distances of genes from *Igλ* in germinal center and naïve B cells. (A and C) Scatter plots showing all distances (µm) measured from the gene marked on the x-axis and the nearest *Igλ* allele in (A) splenic GC B cells 10 days post-immunization with NP-CGG or (C) splenic naïve B cells collected from the same spleen samples as those used in (A). (B and D) Bar graphs summarizing data from (A) and (C) respectively. Data are presented as in Figure 1.

*Igh* is not the only mutating Ig gene in GC B cells. In this system, the GC B cells responding to NP also express *Igλ*, which mutates and could affect mutation of nearby non-Ig genes. We therefore measured the distance from the various genes of interest to the nearest *Igλ* allele. Since *Igλ* and *Bcl6* are both on chromosome 16, 4.6 Mb away from one another, we did not measure the distance between *Bcl6* and *Igλ*. We found that, while deaminated genes were on average closer to *Igλ* than was *β2* *m*, they were not significantly closer than *Mef2b* (Figure 3A, B). This was also true for *Igλ*-*Igh* distances. Therefore, as was the case with distances to *Igh*, we do not observe a correlation between the level of AID targeting of a non-Ig gene and its distance to *Igλ*. This was true in both GC B cells (Figure 3A and 3B, Tables S8 and S9) and naïve B cells (Figure 3C and 3D, Tables S10 and S11).

### Analysis of Human *MYC* Transgenic Mice

The data above show that there is no obvious relationship between the distance to a mutating Ig gene and AID targeting of a non-Ig gene. We therefore considered two alternative hypotheses: the positioning of a gene at a particular location on a particular chromosome influences its mutation/deamination potential, or the coding and regulatory sequences intrinsic to each gene determine its mutation/deamination potential and its position on a particular chromosome is not relevant. As an initial test of these hypotheses, we made mice transgenic for human *MYC* and studied the mutability of the human transgene in its new location as compared to the endogenous *c-Myc* gene. We predicted that if the second model is correct then as long as all relevant coding and regulatory elements are included on the transgene, the transgene should be targeted by AID regardless of the transgene integration site. We chose human *MYC* to allow easy discrimination from mouse *c-Myc* in sequencing and expression analyses and we chose a 94 kb bacterial artificial chromosome (BAC) with 34 kb of sequence upstream of *MYC* and 55 kb downstream to increase chances of including all relevant transcriptional regulatory elements. Given that human *MYC* is mutated in an AID-dependent fashion in some human B cell lymphomas [61] and undergoes recurrent chromosomal translocations with *IGH* [32], [33], it, like mouse *c-Myc*, is likely a target of AID in GC B cells.

Microinjection of the *MYC* BAC yielded two founders (Myc31 and Myc24), both of which contained the entire *MYC* gene along with 55 kb of downstream sequence and at least 21 kb of upstream sequence (data not shown). By Southern blot, we estimated that the Myc31 line contained 2 copies of the transgene and the Myc24 line contained 4 copies (Figure S3). Since we did not know where the Myc31 and Myc24 transgenes had integrated, we performed a 3D-FISH analysis of each transgene relative to *Igh*, *Igλ*, *Igκ*, and endogenous *c-Myc*. This confirmed that all copies of the transgene were integrated in one location in each line, and that neither transgene integration site was linked to endogenous *c-Myc* or any of the Ig genes (Figure S4). In GC B cells, the median distance from Myc31 to *Igh*, *Igλ*, *Igκ*, and endogenous *c-Myc* was 2.62, 2.64, 2.55, and 2.42 µm, respectively, while for Myc 24, these numbers were 2.73, 3.03, 3.11, and 2.67 µm (Figure S4, Tables S12 and S13).

**Figure 4:**
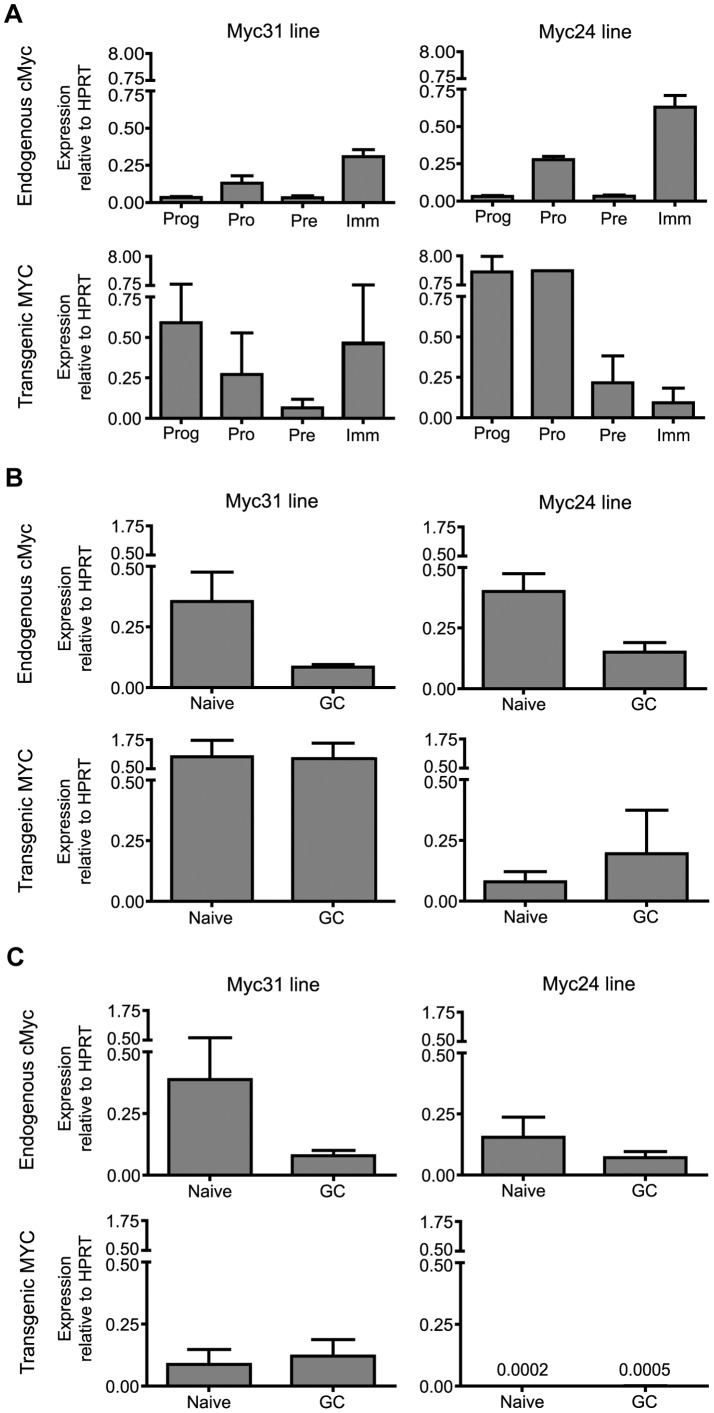
Expression of endogenous *c-Myc* and transgenic *MYC* in B cells. (A, B, and C) Bar graphs represent the average expression level of endogenous *c-Myc* (top row of each panel) or transgenic *MYC* (bottom row of each panel) relative to *Hprt* expression in the same sample for either the Myc31 mouse line (left column in each panel) or the Myc24 line (right column in each panel). Error bars denote the standard error of the mean (SEM). Cell populations analyzed were: (A) Progenitor cells (B220^−^, CD43^−^, IgM^-^), Pro B cells (B220^+^, CD43^+^, IgM^-^), Pre B cells (B220^+^, CD43^−^, IgM^-^), and Immature B cells (B220^+^, CD43^−^, IgM^+^) sorted from bone marrow cells of 4–5 week old mice; (B) Naïve (B220^+^, NIP^lo^, FAS^-^) and GC B cells (B220^+^, NIP^hi^, FAS^+^) sorted from spleens of mice immunized 16 days previously with NP-CGG; and (C) Naïve B cells (B220^+^, CD19^+^, PNA^lo^), and GC B cells (B220^+^, CD19^+^, PNA^hi^) sorted from Peyer’s patches isolated from mice that had lived at least 1 month with non-autoclaved food and tap water. Data represent an average of 2–6 experiments: Panel A top row, 4; panel A bottom row, 2; panel B top row, 6; panel B bottom row, 3; panel C top left, 6; panel C top right, 4; panel C bottom left, 5; panel C bottom right, 3.

Because SHM requires transcription of its target genes [9], we performed qRT-PCR to measure the relative transcription levels of endogenous *c-Myc* and human *MYC* in various B cell samples from bone marrow (Figure 4A), spleen (Figure 4B), and Peyer's patch (Figure 4C). We measured the expression in multiple tissues because we wanted to assess not only the expression levels but also expression patterns in B cell subsets, with the expectation that if all important transcriptional control elements were contained in the BAC, then the expression pattern of the human *MYC* transgene would resemble that of endogenous *c-Myc*.

The expression pattern of endogenous *c-Myc* was similar in Myc24, Myc31, and non-transgenic littermates, demonstrating that the presence of the transgene did not affect the pattern or level of endogenous *c-Myc* expression (data not shown), and data from transgenic and non-transgenic littermates were therefore averaged together in the analyses of *c-Myc* expression of Figure 4. In bone marrow, the highest level of *c-Myc* expression was found in immature B cells, followed by pro-B cells, and there was very little expression in progenitor and pre-B cells. In both spleen and Peyer's patch, expression was higher in naïve B cells than it was in GC B cells, in keeping with a previous analysis that showed that GC B cells express only low levels of *c-Myc* [60].

Both *MYC* transgenes were expressed in bone marrow and splenic B cells, but only Myc31 was expressed at detectable levels in Peyer's patch B cells (Figure 4). In all tissues, transgene expression was deregulated relative to endogenous *c-Myc*. In bone marrow, expression of Myc31 was highest in progenitor and immature B cells and expression of Myc24 was highest in progenitor and pro-B cells. Myc31 and Myc24 in spleen and Myc31 in Peyer's patch are expressed in GC B cells at levels at least as high as in naïve B cells. The deregulation of the transgenes might be caused by a number of factors including the absence of important transcriptional regulatory sequences in the BAC, the change in position of the gene, or the fact that the transgene is the human version of *c-Myc*.

**Figure 5:**
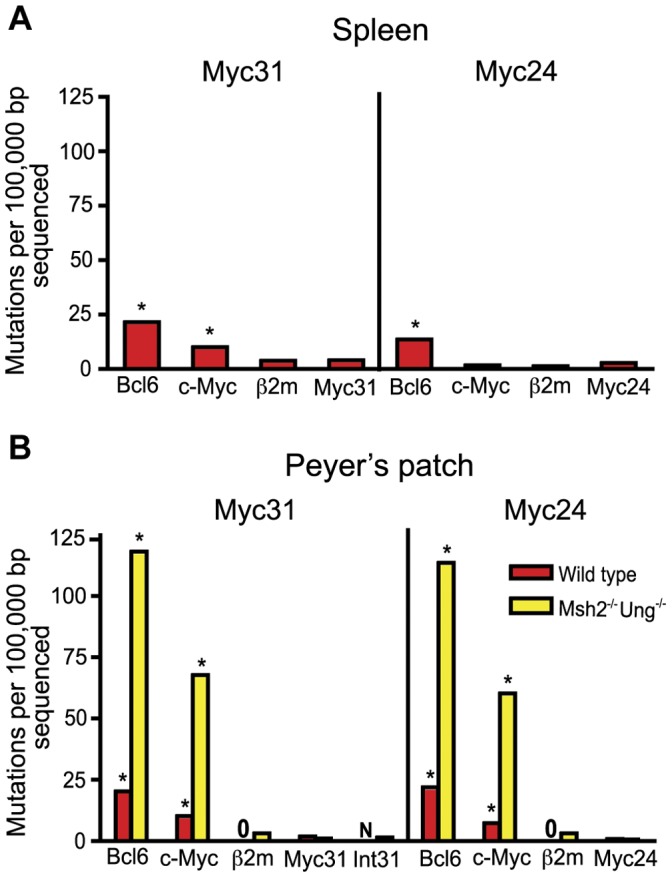
Mutation level in Myc31 and Myc24 transgenic mice. Bar graphs show mutations per 100,000 basepairs sequenced for various non-immunoglobulin genes in germinal center (GC) B cells of *MYC* transgenic mice. Data from the Myc31 line is shown on the left of each graph and the Myc24 line on the right of each graph. Sequence data from the first 1 kb downstream of the MYC P0 promoter is labeled Myc31 or Myc24. Sequence data from the first intron (including the end of the first exon) was done only in Msh2^−/−^Ung^−/−^ dko Myc31 mice and is labeled Int31. A star above the bar indicates that the mutation frequency is significantly (p<0.05) above the background frequency of 1.6 mutations per 100,000 basepairs sequenced reported by Liu et al. [14] using a Chi-square test with Yate’s correction. A “0” in place of a bar indicates that no mutations were found in all sequences analyzed. An “N” in place of a bar indicates that the sample was not sequenced. (A) Splenic GC B cells sorted from Myc31 or Myc24 positive, B1-8 heterozygous mice immunized 16 days prior with NP-CGG. (B) Mutation frequency in Peyer’s patch GC B cells isolated from either wild type mice (red bars) or *Msh2* ^−/−^ *Ung* ^−/−^ double knockout mice (yellow bars) aged at least four months with non-autoclaved food and tap water.

Given that the *MYC* transgenes were transcribed in at least some GC B cell populations, it was possible that they would also be targeted by AID and perhaps accumulate mutations. To examine this, we studied the mutation of Myc31 and Myc24 in splenic GC cells and Peyer's patch GC cells on a WT or *Msh2^−/−^Ung^−/−^* background. We sequenced a region 1 kb downstream of the *MYC* P0 promoter, including more than 400 bp of the first exon. We first performed sequence analysis of the transgenes on the WT background in GC B cells from both spleen and Peyer's patch (Figure 5, red bars, Tables S14, S15, S16, and S17). As expected [14], *Bcl6* had the highest mutation frequency of the non-immunoglobulin genes analyzed, and *β2* *m* did not mutate. We also confirmed very high mutation frequencies at Ig loci (either VJλ1 or the intron downstream of the *Igh* Jh4 gene) in these cells, demonstrating that they had extensive exposure to the SHM machinery (Tables S14, S15, S16, and S17). Endogenous *c-Myc* mutated at a low frequency but was above background in some cases, consistent with a previous analysis of *c-Myc* mutation in Peyer's patch GC B cells [14]. The *MYC* transgenes, however, did not mutate at a significant frequency in either spleen or Peyer's patch WT GC B cells (Figure 5), with a total of ≈550,000 nt of MYC sequence analyzed (Tables S14, S15, S16, and S17).

In a previous study, endogenous *c-Myc* was observed to mutate at a low level in WT GC B cells and its targeting by AID only became obvious when it was sequenced from *Msh2^−/−^Ung^−/−^* double knockout GC B cells, where the mutation frequency was increased almost 20-fold over that seen in WT GC B cells [14]. To determine whether this was also the case for the *MYC* transgenes, we bred Myc31 and Myc24 onto an *Msh2^−/−^Ung^−/−^* background and measured mutation in Peyer's patch GC B cells (Figure 5B, yellow bars, Tables S18 and S19). *Bcl6, c-Myc*, and the *Igh* Jh4 intron accumulated a high mutation load in both the Myc31 and Myc24 lines of mice, while *β2* *m* was not mutated at significant levels, consistent with previous findings [14]. Virtually all mutations observed were C-to-T or G-to-A transition mutations (data not shown), as expected for AID-mediated mutation in the *Msh2^−/−^Ung^−/−^* background [62]. Hence, the GC B cells analyzed were exposed to significant levels of AID-mediated cytidine deamination in the absence of repair. Despite this, neither the Myc31 nor the Myc24 transgene accumulated significant levels of mutation (Figure 5B). Given the lack of expression of Myc24 in Peyer's patch GC B cells (Figure 4C), we expected that it would not mutate. The lack of mutation of the Myc31 transgene, however, was notable because this transgene was expressed about as well as endogenous *c-Myc* in Peyer's patch GC B cells (Figure 4C). Only two Myc31 mutations were detected in over 173,000 nucleotides sequenced (Table S18), yielding a mutation frequency (1.1×10^−5^ mut/nt) that is below the background frequency defined in *AID^−/−^* GC B cells (1.6×10^−5^ mut/nt). To confirm the lack of mutation of Myc31 on the *Msh2^−/−^Ung^−/−^* background, we sequenced a different 1 kb region of this transgene that included the end of the MYC first exon and most of the first intron, the region where mutation frequencies are expected to be the highest [14], [61]. Again we found a total of only three Myc31 mutations in over 192,000 bp sequenced from four independent Peyer's patch GC B cell samples, resulting in a mutation frequency of 1.56×10^−5^ mutations/bp sequenced (Figure 5B, sample labeled “Int31”; Table S18, gene labeled “huMyc31 intron”). We conclude that the *MYC* transgenes were not deaminated by AID despite substantial levels of deamination of other sites in the genome.

## Discussion

### Proximity of Non-Ig genes to Ig Loci does not Correlate with Deamination by AID

Our study of the nuclear localization of genes known to be targeted by AID in GC B cells has revealed no correlation between proximity to Ig genes and mutation. Even the two most highly mutating loci in these GC B cells (*Igλ* and *Igh*) are no closer to one another, on average, than they are to the other loci examined. The simplest and most likely explanation for our results is that proximity to Ig genes does not contribute to a gene's likelihood of being mutated or deaminated by AID in normal mouse GC B cells. This conclusion fits well with recent data indicating that genes that acquire binding of replication protein A (RPA) as a result of the action of AID do not show a tendency to be in close proximity with *Igh*, as measured by chromosome conformation capture experiments [63].

Our data, however, do not rule out all scenarios in which proximity to Ig genes contributes to AID targeting or SHM. We cannot exclude the possibility that the window of proximity to Ig genes and mutation is either before or after the 10 days post-immunization time-point studied, nor can we rule out the idea that a regulated process moves non-Ig genes into close proximity of Ig genes at specific time points during the GC response. We did confirm that the median distance between *c-Myc* and *Igh* is relatively constant from day 8–16 post-immunization and we observed that that the distributions of distances from each non-Ig gene to Ig lociare nearly identical. If the average relative location of other genes is as stable as it is for *c-Myc*, the window of proximity would need to be very early or late in the GC response, or transient, for us to have missed the correlation.

A more likely scenario by which proximity to Ig loci could play a role in mutation or deamination is if a second factor, superimposed on proximity, determines the mutability of a gene. Our data are notable in that, no matter what the gene pair analyzed, the dataset contains at least a few close associations between the Ig and non-Ig gene. It is possible that deamination/mutation is most likely to occur on these alleles, but since all datasets contain such associations, it is clear that low frequency proximity to Ig loci is not sufficient to render a gene mutable. However, our data are compatible with a requirement for proximity if a second factor, such as E boxes in the vicinity of the transcription start site, also contributes to mutability of a gene. Indeed, E boxes were the only transcription factor binding motif found to be enriched near the promoter in the most mutated group of non-Ig genes in the Liu et al. study [14]. Further study of SHM and the factors affecting it will elucidate whether proximity works together with other mechanisms to determine mutability. Our finding that all non-Ig genes analyzed come into close proximity with the Ig loci analyzed in a small percentage of cells is consistent with recent studies demonstrating the ability of the *Igh* locus to engage in chromosomal translocations with many other sites in the genome [63], [64], [65], [66]. Of particular relevance is the finding that eight different chromosomal sites interacted with and engaged in frequent chromosomal translocations with five different antigen receptor loci in pre-B cell lines undergoing V(D)J recombination [66]. The results of these studies and others strongly suggest that the organization of chromosomes in the nucleus is heterogeneous and that many (perhaps most) pairs of expressed regions in the genome have a meaningful probability of coming into close proximity [67].

We expect that our static picture of gene position in fixed cells is a good representation of the distribution of gene arrangements in live GC B cells. Although genes may loop out of their territories [68], [69], [70] and in some cases chromosomes may be randomly arranged in very early G1 phase [71], it appears that mammalian chromosome territories are relatively stable and chromatin does not diffuse widely [72], [73]. While we cannot rule out the possibility that genes move during purification and sorting for 3D-FISH after the cells are removed from the GC, we tried to minimize changes in gene position by following a methodology that maintains nuclear architecture and spherical nuclei. One limitation of our approach is revealed by recent studies that show that B cells change shape significantly as they squeeze through small spaces during movement in the GC [74], [75]. Such dynamic cellular deformations might alter intergenic spacing and would presumably not be captured by our analysis.

This study assessed whether mutation of a gene is determined by the closeness of that gene to one of the mutating Ig genes. It did not, however, assess other ways that nuclear localization could play a role in mutation. It is possible that there are mutation hubs for non-Ig genes but the hubs do not center around an Ig gene. It is also possible that certain non-Ig genes have a propensity to be near an Ig gene at an early time point prior to GC formation and AID expression, as is the case for *c-Myc* and *Igh* in ex vivo activated B cells [54]. Such genes could be marked for deamination/mutation at that early time point and mutate later when AID is expressed.

### Position of *c-Myc* Relative to *Igh*

Genes that translocate frequently with one another have been shown in other studies to have a tendency to be close to one another [54], [76], [77], [78]. It is known that *c-Myc* translocates with *Igh*, *Igλ*, and *Igκ*. One study found that *c-Myc* moved closer to *Igh* immediately after ex vivo activation [54] and we were able to replicate this result. Another study of ex vivo activated B cells did not find *c-Myc* and *Igh* nearer to one another after activation, but did find that *c-Myc* is closer to *Igh* in B cells than it is in ES cells [79]. Furthermore, a study of *MYC* relative to all three Ig loci in a human B cell line found that it is closer to *IGL* and *IGH*, to which it translocates most, than it is to *IGK* [80].

These data, together with the results of two recent genome-wide translocation analyses [63], [66], are consistent with a model in which proximity contributes to translocation frequency. Because *MYC-IG* translocations are a common feature of GC derived lymphoid tumors in humans, it is important to understand the mechanisms that lead to these translocations. One possibility is that they are favored by close proximity between *MYC* and Ig loci in GC B cells. Our data provide the first direct test of this idea, and show that, contrary to this simple expectation, *c-Myc* is actually the most distant from *Igh* of the seven loci tested in GC B cells (Figure 1C and 1D) and is not significantly closer to *Igh* than the other genes tested in naïve B cells (Figure 1E and 1F). Our study of non-Ig genes relative to *Igλ* gave a similar result; in both GC and naïve B cells *c-Myc* was as far or farther from *Igλ* than most of the other loci examined (Figure 3).

Hence, we have no evidence that *c-Myc-Igh* or *c-Myc-Igλ* translocations might be especially favored in GC B cells due to unusually close spatial proximity between *c-Myc* and these Ig loci. This is consistent with the recent finding that *c-Myc* and *Igh* do not come into close physical proximity at a notably high frequency in ex vivo activated splenic B cells [63]. Indeed, although we found *c-Myc* to be significantly closer to *Igh* and *Igλ* than it is to *Igκ* in naïve B cells, consistent with previous work on a human B cell line [80], we observed that this difference is lost in GC B cells (Figure S5, Table S20, S21, S22, and 23). However, our data do not preclude a requirement for close proximity for translocation. We do detect very close proximity between *c-Myc* and Ig loci in a small fraction of GC B cells and it is possible that *c-Myc*-Ig translocations are favored in this subset of cells. Also, as noted above, others have found that *c-Myc* is closer to *Igh* in B cells than in non-B cells [79], a comparison we have not made, and this might enhance the probability of *c-Myc-Igh* translocations in the B lineage.

It remains to be determined whether *c-Myc-Igh* or *c-Myc-Igλ* translocations occur with a particularly high frequency in GC B cells. Relevant to this is the finding that when *c-Myc* coding sequences are replaced by those of *N-myc*, *N-myc* becomes a frequent *Igh* translocation partner in pro-B cell lymphomas in mice lacking Ligase 4 and p53 [53]. This result argues that there are mechanistic features of the *c-Myc* locus that predispose it to translocate with *Igh* in pro-B cells. It is possible that there is a similar predisposition of *c-Myc* to translocate with Ig loci in GC B cells, although clear evidence for this is lacking. The appearance of *MYC*-Ig translocations in GC derived B cell tumors could simply be the result of strong cellular selection. In this regard, two recent genome-wide studies failed to detect *c-Myc* as a hotspot for translocation with a defined DSB in the *Igh* locus in ex vivo activated B cells expressing physiological levels of AID [64], [65], although one study found *c-Myc* to be a translocation hotspot when AID was overexpressed [65]. We propose that if *c-Myc* is a favored translocation partner for *Igh* or *Igλ* in GC B cells, this is due to features of the *c-Myc* locus other than an unusually close proximity to these Ig loci. An obvious predisposing feature would be the fact that *c-Myc* is targeted by AID.

### Mutation and Expression of Human *MYC* Transgenes

To assess whether the chromosomal location of *MYC* affects its mutation we made mice transgenic for human *MYC*. If *MYC* contains intrinsic sequence features that render it a good AID target, then it should be a good AID target as long as it is expressed no matter where it integrates in the genome. If, however, the particular genomic location of endogenous *MYC* is crucial for AID targeting, then it might not be targeted in other locations. Mutation and expression analysis on two *MYC* transgenic mouse lines indicated that *MYC* is not mutated or even deaminated in two new locations despite the fact that it is expressed in splenic GC B cells and, in the case of Myc31, also expressed in Peyer's patch GC B cells.

From this we conclude that the sequences contained in the *MYC* BAC used in our experiments do not contain sufficient information to target AID to the transgene in mouse GC B cells. There could be several explanations for this. First, it might be that the position of the *MYC* locus in the genome is critical for AID targeting and this positioning is lost in the *MYC* transgenes. Second, the human *MYC* locus might contain intrinsic sequence features critical for AID targeting, but those sequence features were either not included in the BAC transgene or were not able to exert their activity in mouse GC B cells. The failure of the *MYC* transgenes to be expressed in a pattern that mimics endogenous *c-Myc* strongly suggests that important transcriptional regulatory elements were missing from the BAC or did not function properly in mouse GC B cells. Transgenic mice created in another lab using a rat BAC spanning the *c-Myc* locus also resulted in deregulated transgene expression relative to the endogenous *c-Myc* gene (R. Casellas, personal communication). The fact that *c-Myc* BACs, even from rat, which is more closely related to mouse than is man, were unable to express normally makes the importance of distant elements or nuclear positioning more likely. Third, we cannot rule out the possibility that human and mouse *MYC* loci obey different rules in relation to AID targeting. Mouse *c-Myc* is one of the strongest known non-Ig gene targets for AID in normal GC B cells, although few mutations accumulate due to high-fidelity repair [14]. Because the breakpoints in human *MYC* translocations often lie in its first exon or intron, precisely where AID would be expected to be most active [61], it is reasonable to think that *MYC* is also a strong AID target in normal human GC B cells. This, however, remains to be directly demonstrated. Previous sequencing of *MYC* in human peripheral memory B cells showed no significant level of mutation [10], [81], but it is possible that human *MYC*, like mouse *c-Myc*, is primarily repaired in a high-fidelity manner. It is worth noting that *MYC* is significantly mutated by AID in certain human B cell lymphomas [61].

In summary, while our data argue against a role for proximity of non-Ig genes to Ig loci in determining AID targeting or the accumulation of mutations in mouse GC B cells, they do not support strong conclusions regarding the question of whether the genomic location of human *MYC* (and by extension, mouse *c-Myc*) contributes to its being a strong target of AID and a participant in chromosomal translocations.

## Materials and Methods

### Ethics Statement

All mouse work in this study was performed in accordance with the Guide for the Care and Use of Laboratory Animals of the National Institutes of Health. All procedures were approved by the Institutional Animal Care and Use Committee of Yale University (protocol number 2009-07610) and every possible effort was made to minimize suffering.

### Generation of Transgenic Mice

Transgenic mice were made by microinjection of embryos with BAC CTD-3056O22 (Invitrogen), which contains the human *MYC* gene. BAC DNA was isolated using a Qiagen large construct kit and then linearized overnight and run on a pulse field gel in 0.9% low melting point agarose (Seaplaque) in TAE. The BAC DNA was then purified from the gel by melting, digesting overnight with 10 u β-agarase, spinning to remove large particles, and separating larger DNA fragments using a Millipore ultrafree-MC filter with a 30 kD molecular weight cut off. The DNA was microinjected at the Yale Animal Genomics Services facility. Potential founders were screened first by PCR and then confirmed by Southern blot. Founders were analyzed by PCR to determine how many kb of upstream and downstream sequence had integrated as part of the transgene. The two transgenic mouse lines were maintained in mouse facilities at Yale Medical School by mating with Balb/c WT mice from Jackson Laboratories. Transgene positive mice were also bred with B1-8 knockin mice to generate B1-8 heterozygous Myc transgene positive mice. Each transgenic line was also crossed with Msh2*^+/−^*Ung^−/−^ mice and offspring were bred to generate Myc24*^+^*Msh2^−/−^Ung^−/−^ and Myc31*^+^*Msh2^−/−^Ung^−/−^ mice. All primers used for Southern blot probes, typing, and determination of BAC integration are available upon request.

### 3D-FISH

For each experiment, five B1-8 knockin heterozygous mice were immunized intraperitoneally with 25 µg NP-CGG precipitated in alum. 10 days later spleens were removed and red blood cells were lysed. Some of the cell suspension was saved for sorting of naïve B cells. From the rest, GC B cells were negatively selected using a custom order Easysep kit for B cell enrichment without α-CD43 (Stemcell). 1∶800 α-CD38 (BD cat# 553762) conjugated to biotin was added with the antibody mix provided by Stemcell. Cells were stained with NIP conjugated to PE (conjugated in lab), α-FAS (CD95) PE-Cy7 (BD cat# 557653), and α-B220 A700 (BD cat# 557957), sorted, and used immediately for 3D-FISH. BACs to be used for fluorescent probes were prepared using a Qiagen large construct kit and then labeled using a nick translation mix (Roche cat# 11745808910) and either A488-5-dUTP (Invitrogen cat# C-11397), A568-5-dUTP (Invitrogen cat# C-11399), or Cy5-dUTP (GE healthcare cat# PA55026). All BACs used for 3D-FISH are listed in Table S23. The nick translation reaction was allowed to progress at 16°C until most of the DNA was 100-600 bp in size. Probes were filtered for 2 hours on 0.025 mm filters (Millipore cat# VSWP02500), and precipitated with 9.9 µg salmon sperm DNA, 0.9 µg mouse Hyblock DNA, and 0.9 µg COT1 DNA for every 1 µg probe DNA. All probes to be used on a slide were precipitated together, resuspended in a dextran sulphate hybridization mix, heated at 95°C for 5 minutes, and then left at 37°C for about 1 hour until hybridization. Interphase FISH was done as previously reported [82]. Briefly, cells attached to poly-L-lysine coated coverslips were put in various solutions: 0.1% triton X and 0.5% glutaraldehyde for 30 minutes, 4% (w/v) sodium borohydride for 2×15 minutes, PBS with 5% FBS and 5% NGS for 30 minutes, ethylene glycol bis(succinimidyl succinate) for 30 minutes at 37°C, and 100 µg/ml RNaseA in 2X SSC for 1 hour. The coverslips were then put in 1N NaOH for exactly 2 minutes, washed in ice cold PBS, and hybridized with probe overnight at 37°C in a wet chamber. After hybridization coverslips were washed in SSC and mounted on slides with Vectashield with DAPI.

### Microscopy and Analysis of FISH Data

FISH slides were analyzed using a Leica SP5 scanning confocal microscope with a 63X 1.4 oil lens. Scans were done in one direction at a speed of 400 Hz and 4 frames were averaged for each XY picture. In Z, a picture was taken every 0.18 µm to create a Z stack. The software Volocity was used to find the nuclei and probe spots and to obtain the X, Y and Z centroid coordinates of each. The Volocity output was analyzed with a homemade Stata program that calculated the distances between each of the centroids associated with one cell. Kolmogorov-Smirnov tests were done on the website http://www.physics.csbsju.edu/stats/KS-tests.html.

### Expression Analysis

#### Peyer's patch

Peyer's patches were removed from two to four euthanized mice that had been maintained on non-autoclaved food and water. Germinal center B cells were sorted based on staining with B220-cychrome (BD), CD19-PE (BD), and PNA-FITC (Vector Labs). The B220^+^, CD19^+^ and PNA lo naïve B cells were also sorted. **Bone marrow:** Bone marrow cells were collected from the leg bones of euthanized 4–5 week old mice. Red blood cells were lysed and the remaining cells were stained with α-B220-PerCP, α-CD43-PE, and α-IgM-APC (all from BD) and four populations were sorted: Progenitor (B220^−^, CD43^−^, IgM^−^), Pro B (B220^+^, CD43^+^, IgM^-^), Pre B (B220^+^, CD43^−^, IgM^−^), and Immature B (B220^+^, CD43^−^, IgM^+^) cells. **Spleen:** Spleens were removed from two euthanized mice, mashed, and red blood cells were lysed. The white blood cells were separated using a MACS kit for depletion of non-B cells and the B cell fraction was resuspended in B cell media and activated with LPS and IL-4 for 2–3 days. **All samples:** RNA was prepared from cells using the Qiagen RNA isolation kit and Qiashredder. The eluate was rerun through the same column to increase yield. RNA concentration was measured using a NanoDrop spectrophotometer. RNA was treated with DNase (Invitrogen) and then used to make cDNA with Superscript II RT (Invitrogen), random hexamer primers, and RNaseOUT (Invitrogen). Approximately equal amounts of RNA were used for each reaction. qPCR was done in duplicate with primers spanning a large intron and a TaqMan 5'FAM/3'BHQ-1 probe (Biosearch) complementary to the expected product. All initial concentrations were calculated by comparison to a previously tested gene specific standard. 25 µl reactions using Qiagen HotStarTaq were allowed to proceed for 40 cycles on a Stratagene MX3000P thermal cycler.

### Sequence Analysis

Peyer's patch B cell sorts for sequence analysis were done as for expression analysis except that mice were aged with non-autoclaved food and water for at least 4 months. We were also able to sort Peyer's patch B cells from only one to three mice per group since the Peyer's patches were usually larger in both size and number in the aged mice. Spleen sorts for sequence analysis were done as for FISH except that spleens were removed on day 16 post-immunization. The preparation of DNA, PCR amplification and TOPO cloning were all done as previously reported [14]. Clones were picked into 96 or 384 well plates and sent for sequencing at Beckman Coulter. Sequence analysis was performed as reported [14] except that redundant mutations were not scored.

## Supporting Information

Figure S1 **Representative FACS set up for collecting splenic B cells.** Live cells were selected in the side scatter (SSC-A) vs. forward scatter (FSC-A) plot (A). Doublets and clumps were excluded in the side scatter width (SSC-W) vs. side scatter height (SSC-H) and forward scatter width (FSC-W) vs. forward scatter height (FSC-H) plots (B). Cells were gated for B220 expression (C), as well as FAS expression, and NIP binding (D). Germinal center B cells (FAS^+^NIP^+^) were sorted from spleen samples that had been magnetically separated to remove non-B cells and naïve B cells. Naïve B cells (FAS^-^NIP^-^) were sorted from unmanipulated spleen samples.
(PDF)

Figure S2 **Mutation frequencies in GC B cells from spleen and Peyer’s patch.** Bars represent mutations per 100,000 basepairs sequenced from various non-immunoglobulin genes in germinal center (GC) B cells. The actual number of mutations found in each gene is shown in parentheses above each bar. A star above the bar indicates that the mutation frequency was determined to be significantly (p<0.05) above the background frequency of 1.6 mutations per 100,000 basepairs sequenced reported by Liu et al. [14] using a Chi-square test with Yate’s correction. (A) Splenic GC B cells were sorted from B1-8 heterozygous (het) mice immunized 16 days prior with 4-hydroxy-3-nitrophenylacetyl conjugated to chicken gamma globulin (NP-CGG). (B) Mutation frequencies in Peyer’s patch B cells as reported in Liu et al. [14].
(PDF)

Figure S3 **Southern blot of transgenic mouse DNA to confirm the presence of the human** ***MYC*** **transgene and estimate copy number.** Myc24^+^ (24+), and Myc31^+^ (31+) DNA were compared to human DNA (hum) and non-transgenic littermate DNA (24 LM and 31 LM), as indicated above the lanes. The agarose gel of the BglII digested DNA is shown on the right, and the hybridized blot is shown on the left. The 5 and 6 kb marker bands of the agarose gel are indicated. Two identical southern blots were analyzed and Myc24 was estimated to contain 4 copies of the transgene and Myc31 was estimated to contain 2 copies.
(PDF)

Figure S4 **The distance from the huMyc31 or huMyc24 transgenes to Ig genes and endogenous** ***c-Myc*** **.** Scatter plots are presented showing all distances (µm) measured from the (A) Myc31 or (B) Myc24 transgene to the nearest allele of the gene marked on the x-axis. The number of measurements taken is shown in parentheses above each data set and red bars mark the median. Data are presented as in Figure 1.
(PDF)

Figure S5 **The distance from** ***c-Myc*** **to Ig loci in germinal center and naïve B cells.** Scatter plots show all distances (µm) measured from *c-Myc* to the nearest allele of the gene marked on the x-axis in splenic GC or naïve B cells 10 days post-immunization with NP-CGG. Data for *c-Myc* relative to *Igh* and *c-Myc* relative to *Igλ* are the same as those shown in Figures 1 and 3 respectively. Data are presented as in Figure 1.
(PDF)

Table S1 **Mutation in B1-8 het splenic GC cells.** Table shows the mutations (Mut) per base pair sequenced (bp) for various genes (Gene) in each splenic GC cell sample (Sample). The mutations and number of base pairs sequenced are added together for each gene and the mutation frequency (Frequency) is calculated by dividing the total number of mutations by the total number of base pairs sequenced. A “Yes” in the p<0.05 column indicates that the mutation frequency is statistically above background (p<0.05) as determined by a Chi-square test with Yate’s correction. The background mutation level of 1.6 mutations per 100,000 base pairs sequenced was determined in Liu et al. [14].
(PDF)

Table S2 **Summary of FISH data for genes relative to** ***Igh*** **in GC B cells.** Supporting data for graphs in Figure 1C and 1D. For each gene analyzed relative to *Igh* the number of slides analyzed (Slides), number of measurements taken (Number), median distance (Median), mean distance (Mean), standard deviation (St. Dev.), and 95% confidence interval (95% conf. int.) are shown. Individual slides came from independent experiments. Only cells in which both alleles of both loci of interest were clearly labeled by FISH probes were included in analysis. Two measurements were taken from each cell.
(PDF)

Table S3 **KS tests of FISH data for distance to** ***Igh*** **in GC cells.** Kolmogorov-Smirnov (KS) test results derived from the datasets of Figure 1C and 1E. KS tests, which compare dataset distributions, were done using the website http://www.physics.csbsju.edu/stats/KS-test.html. Datasets on the left were compared with datasets on the top. The p value is shown, and for any comparison that led to a p value less than 0.1, the corresponding D value is shown in parentheses. D is the maximum vertical distance between the two cumulative fraction plots and indicates the magnitude of the difference between the two distributions.
(PDF)

Table S4 **Summary of FISH data for genes relative to** ***Igh*** **in naïve B cells.** Supporting data for graphs in Figure 1E and 1F. See the legend of Table S2 for a full description.
(PDF)

Table S5 **KS tests of FISH data for genes relative to** ***Igh*** **in naïve cells.** KS test results comparing the datasets in Figure 1E and 1F. See the legend of Table S3 for a full description.
(PDF)

Table S6 **Summary of FISH data for** ***c-Myc*** **relative to** ***Igh*** **at various timepoints.** Supporting data for graphs in Figure 2A and 2B. See the legend of Table S2 for a full description.
(PDF)

Table S7 **KS tests of FISH data for** ***c-Myc*** **relative to** ***Igh*** **at various timepoints.** KS test results comparing the datasets in Figure 2A and 2B. See the legend of Table S3 for a full description.
(PDF)

Table S8 **Summary of FISH data for genes relative to** ***Igλ*** **in GC cells.** Supporting data for graphs in Figure 3A and 3B. See the legend of Table S2 for a full description.
(PDF)

Table S9 **KS tests of FISH data for genes relative to** ***Igλ*** **in GC cells.** KS test results comparing the datasets used in Figure 3A and 3B. See the legend of Table S3 for a full description.
(PDF)

Table S10 **Summary of FISH data for genes relative to** ***Igλ*** **in naïve cells.** Number of slides analyzed, number of distances measured, and statistical analysis for each of the datasets included in Figure 3C and 3D. See the legend of Table S2 for a full description.
(PDF)

Table S11 **KS tests of FISH data for genes relative to** ***Igλ*** **in naïve cells.** KS test results comparing the datasets used in Figure 3C and 3D. See the legend of Table S3 for a full description.
(PDF)

Table S12 **Summary of FISH data for Myc31 relative to other genes.** Supporting data for graph in Figure S4A. See the legend of Table S2 for a full description.
(PDF)

Table S13 **Summary of FISH data for Myc24 relative to other genes.** Supporting data for graph in Figure S4B. See the legend of Table S2 for a full description.
(PDF)

Table S14 **Mutation in B1-8 het Myc31^+^ splenic GC cells.** Supporting data for left side of graph in Figure 5A. See the legend of Table S1 for a full description.
(PDF)

Table S15 **Mutation in B1-8 het Myc24^+^ splenic GC cells.** Supporting data for right half of graph in Figure 5A. See the legend of Table S1 for a full description.
(PDF)

Table S16 **Mutation in Myc31^+^ Peyer's patch GC B cells.** Supporting data for red bars in the left half of the graph in Figure 5B. See the legend of Table S1 for a full description.
(PDF)

Table S17 **Mutation in Myc24^+^ Peyer's patch GC B cells.** Supporting data for red bars in the right half of the graph in Figure 5B. See the legend of Table S1 for a full description.
(PDF)

Table S18 **Mutation in Myc31^+^Msh2^−/−^Ung^−/−^ Peyer's patch GC B cells.** Supporting data for yellow bars in the left half of Figure 5B. See the legend of Table S1 for a full description.
(PDF)

Table S19 **Mutation in Myc24^+^Msh2^−/−^Ung^−/−^ Peyer's patch GC B cells.** Supporting data for yellow bars in the right half of the graph in Figure 5B. See the legend of Table S1 for a full description.
(PDF)

Table S20 **Summary of FISH data for** ***c-Myc*** **relative to** ***Igκ***. Supporting data for *Igκ* data in Figure S5. See the legend of Table S2 for a full description.
(PDF)

Table S21 **KS tests of FISH data for** ***c-Myc*** **relative to Ig loci in GC B cells.** KS test results comparing the GC cell datasets in Figure S5. See the legend of Table S3 for a full description.
(PDF)

Table S22 **KS tests of FISH data for** ***c-Myc*** **relative to Ig loci in naive B cells.** KS test results comparing the naïve cell datasets in Figure S5. See the legend of Table S3 for a full description.
(PDF)

Table S23 **BACs used for FISH analysis.** BACs used for FISH analysis are listed (BAC Name) along with their accession numbers (Accession), size in kb (Size), and position (Relative position) with reference to the gene of interest (Gene). The chromosome number on which each gene is found (Chr) is listed, as are the mutation groups of each gene as determined by Liu et al. [14].
(PDF)
