# Supplementary figures and images for: AID-Targeting and Hypermutation of Non-Immunoglobulin Genes Does Not Correlate with Proximity to Immunoglobulin Genes in Germinal Center B Cells
Source: PLoS One. 2012 Jun 29;7(6):e39601. doi: 10.1371/journal.pone.0039601 (PMC3387148; doi:10.1371/journal.pone.0039601)

**A**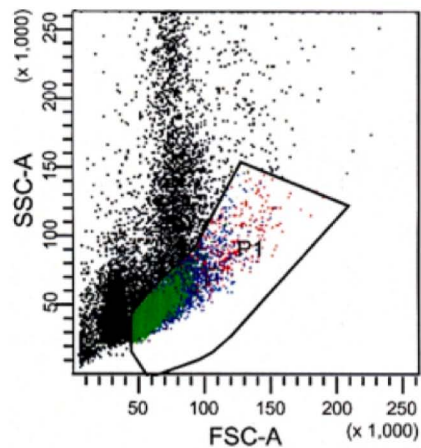**B**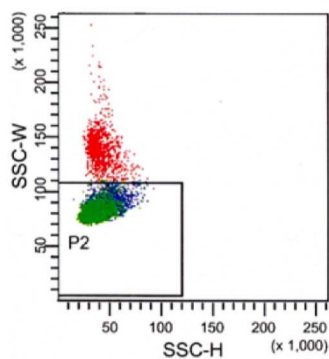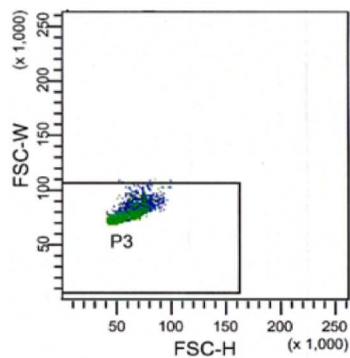**C**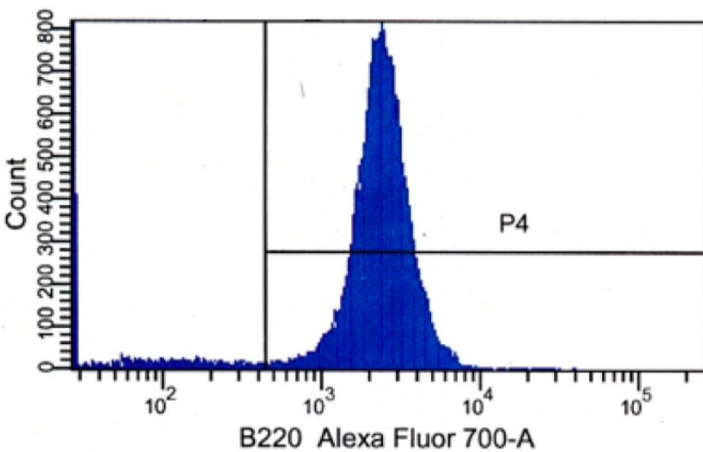**D**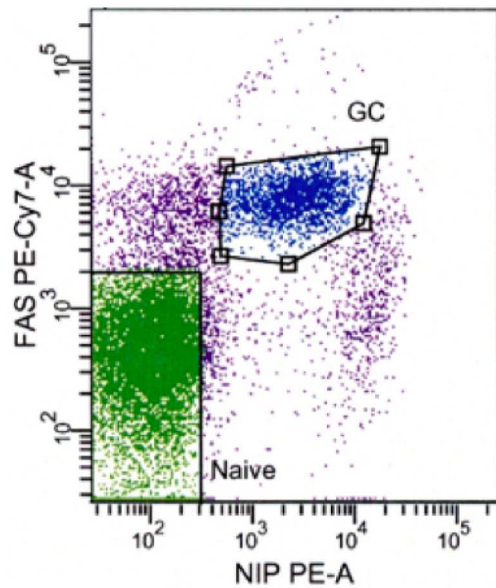

Supplement: Figure S1 — Representative FACS set up for collecting splenic B cells. Live cells were selected in the side scatter (SSC-A) vs. forward scatter (FSC-A) plot (A). Doublets and clumps were excluded in the side scatter width (SSC-W) vs. side scatter height (SSC-H) and forward scatter width (FSC-W) vs. forward scatter height (FSC-H) plots (B). Cells were gated for B220 expression (C), as well as FAS expression, and NIP binding (D). Germinal center B cells (FAS+NIP+) were sorted from spleen samples that had been magnetically separated to remove non-B cells and naïve B cells. Naïve B cells (FAS-NIP-) were sorted from unmanipulated spleen samples. (PDF) [file pone.0039601.s001.pdf]

**A**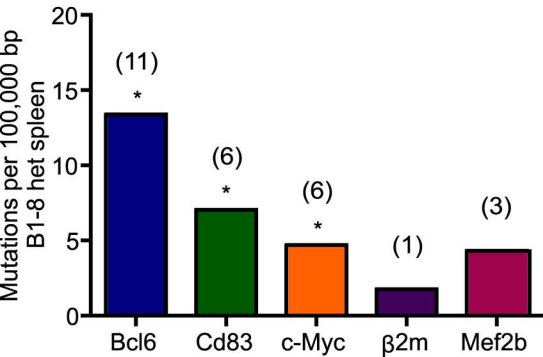**B**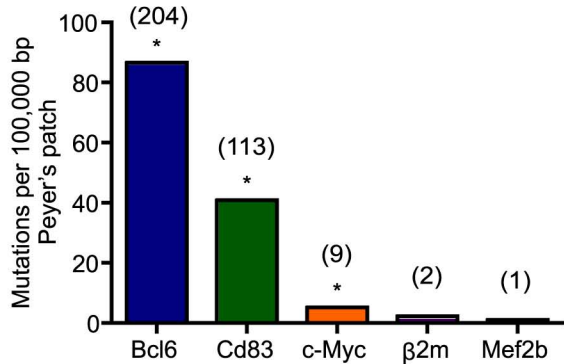

Supplement: Figure S2 — Mutation frequencies in GC B cells from spleen and Peyer’s patch. Bars represent mutations per 100,000 basepairs sequenced from various non-immunoglobulin genes in germinal center (GC) B cells. The actual number of mutations found in each gene is shown in parentheses above each bar. A star above the bar indicates that the mutation frequency was determined to be significantly (p<0.05) above the background frequency of 1.6 mutations per 100,000 basepairs sequenced reported by Liu et al. [14] using a Chi-square test with Yate’s correction. (A) Splenic GC B cells were sorted from B1-8 heterozygous (het) mice immunized 16 days prior with 4-hydroxy-3-nitrophenylacetyl conjugated to chicken gamma globulin (NP-CGG). (B) Mutation frequencies in Peyer’s patch B cells as reported in Liu et al. [14]. (PDF) [file pone.0039601.s002.pdf]

Figure S3

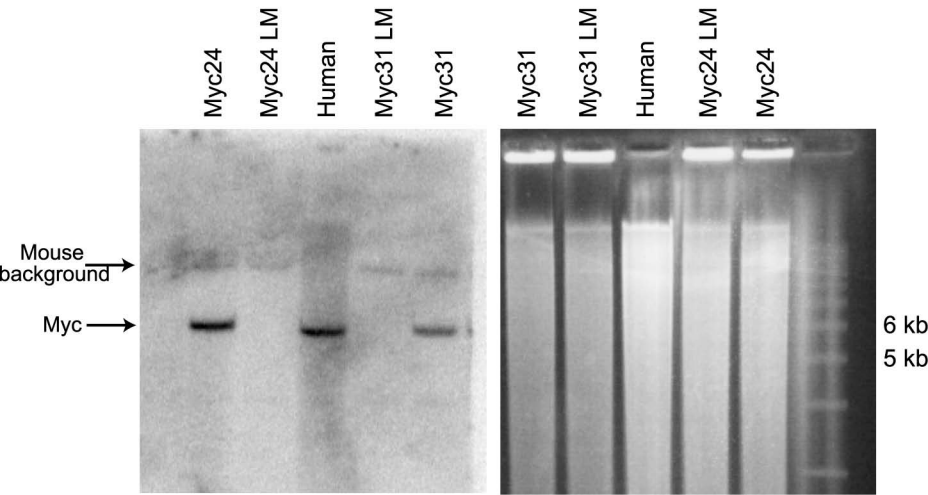

Supplement: Figure S3 — Southern blot of transgenic mouse DNA to confirm the presence of the human MYC transgene and estimate copy number. Myc24+ (24+), and Myc31+ (31+) DNA were compared to human DNA (hum) and non-transgenic littermate DNA (24 LM and 31 LM), as indicated above the lanes. The agarose gel of the BglII digested DNA is shown on the right, and the hybridized blot is shown on the left. The 5 and 6 kb marker bands of the agarose gel are indicated. Two identical southern blots were analyzed and Myc24 was estimated to contain 4 copies of the transgene and Myc31 was estimated to contain 2 copies. (PDF) [file pone.0039601.s003.pdf]

**A**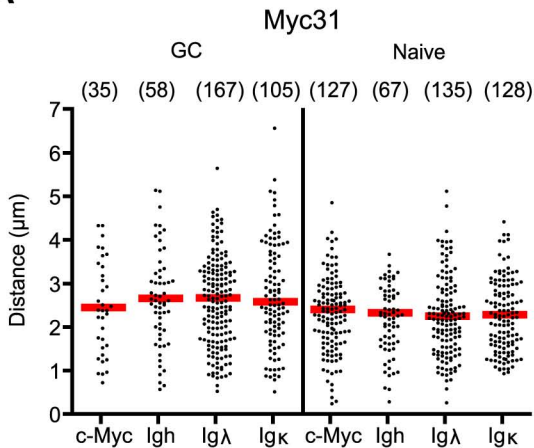**B**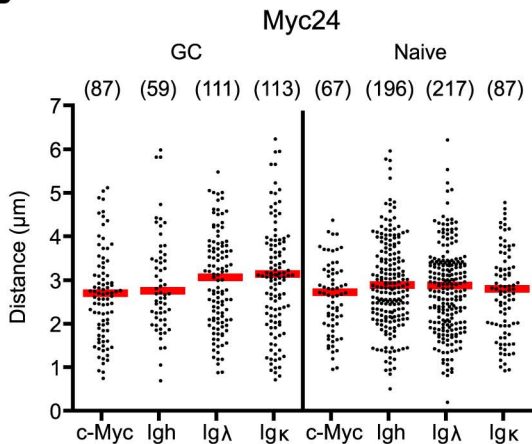

Supplement: Figure S4 — The distance from the huMyc31 or huMyc24 transgenes to Ig genes and endogenous c-Myc . Scatter plots are presented showing all distances (µm) measured from the (A) Myc31 or (B) Myc24 transgene to the nearest allele of the gene marked on the x-axis. The number of measurements taken is shown in parentheses above each data set and red bars mark the median. Data are presented as in Figure 1. (PDF) [file pone.0039601.s004.pdf]

Figure S5

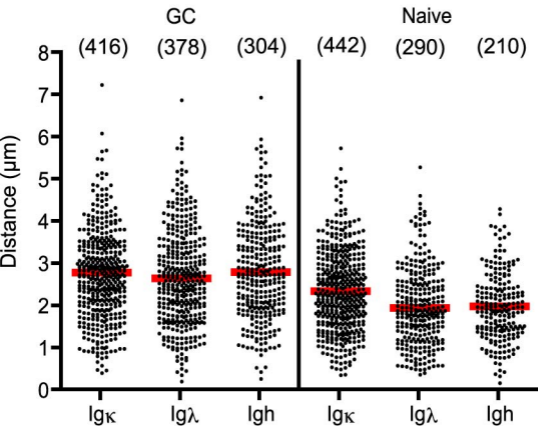

Supplement: Figure S5 — The distance from c-Myc to Ig loci in germinal center and naïve B cells. Scatter plots show all distances (µm) measured from c-Myc to the nearest allele of the gene marked on the x-axis in splenic GC or naïve B cells 10 days post-immunization with NP-CGG. Data for c-Myc relative to Igh and c-Myc relative to Igλ are the same as those shown in Figures 1 and 3 respectively. Data are presented as in Figure 1. (PDF) [file pone.0039601.s005.pdf]
