# Supplementary material for: AID-Targeting and Hypermutation of Non-Immunoglobulin Genes Does Not Correlate with Proximity to Immunoglobulin Genes in Germinal Center B Cells
Source: PLoS One. 2012 Jun 29;7(6):e39601. doi: 10.1371/journal.pone.0039601 (PMC3387148; doi:10.1371/journal.pone.0039601)
Supplement: Table S1 — Mutation in B1-8 het splenic GC cells. Table shows the mutations (Mut) per base pair sequenced (bp) for various genes (Gene) in each splenic GC cell sample (Sample). The mutations and number of base pairs sequenced are added together for each gene and the mutation frequency (Frequency) is calculated by dividing the total number of mutations by the total number of base pairs sequenced. A “Yes” in the p<0.05 column indicates that the mutation frequency is statistically above background (p<0.05) as determined by a Chi-square test with Yate’s correction. The background mutation level of 1.6 mutations per 100,000 base pairs sequenced was determined in Liu et al. [14]. (PDF) [file pone.0039601.s006.pdf]

**Table S1. Mutation in B1-8 het splenic GC cells.**

| Gene | Sample | Mut | bp | Frequency | p<0.05 |
|---|---|---|---|---|---|
| *Bcl6* | 1 | 7 | 53353 | | |
| | 2 | 4 | 28784 | | |
| | total | 11 | 82137 | 13.4 E-05 | Yes |
| *Cd83* | 1 | 4 | 40446 | - | |
| | 2 | 2 | 44795 | - | |
| | total | 6 | 85241 | 7.04 E-05 | Yes |
| *c-Myc* | 1 | 4 | 80123 | - | |
| | 2 | 2 | 47198 | - | |
| | total | 6 | 127321 | 4.71 E-05 | Yes |
| *β2m* | 1 | 0 | 26561 | - | |
| | 2 | 1 | 29941 | - | |
| | total | 1 | 56502 | 1.77 E-05 | No |
| *Mef2b* | 1 | 2 | 34342 | - | |
| | 2 | 1 | 34988 | - | |
| | total | 3 | 69330 | 4.33 E-05 | No |
| *VJλ1* | 1 | 83 | 18204 | - | |
| | 2 | 72 | 16972 | - | |
| | total | 155 | 35176 | 441 E-05 | Yes |

Table shows the mutations (Mut) per base pair sequenced (bp) for various genes (Gene) in each splenic GC cell sample (Sample). The mutations and number of base pairs sequenced are added together for each gene and the mutation frequency (Frequency) is calculated by dividing the total number of mutations by the total number of base pairs sequenced. A "Yes" in the p<0.05 column indicates that the mutation frequency is statistically above background ($p < 0.05$) as determined by a Chi-square test with Yate's correction. The background mutation level of 1.6 mutations per 100,000 base pairs sequenced was determined in Liu et al. [14].
