# Supplementary material for: AID-Targeting and Hypermutation of Non-Immunoglobulin Genes Does Not Correlate with Proximity to Immunoglobulin Genes in Germinal Center B Cells
Source: PLoS One. 2012 Jun 29;7(6):e39601. doi: 10.1371/journal.pone.0039601 (PMC3387148; doi:10.1371/journal.pone.0039601)
Supplement: Table S2 — Summary of FISH data for genes relative to Igh in GC B cells. Supporting data for graphs in Figure 1C and 1D. For each gene analyzed relative to Igh the number of slides analyzed (Slides), number of measurements taken (Number), median distance (Median), mean distance (Mean), standard deviation (St. Dev.), and 95% confidence interval (95% conf. int.) are shown. Individual slides came from independent experiments. Only cells in which both alleles of both loci of interest were clearly labeled by FISH probes were included in analysis. Two measurements were taken from each cell. (PDF) [file pone.0039601.s007.pdf]

Table S2. Summary of FISH data for genes relative to *Igh* in GC B cells.

	Slides	Number	Median	Mean	St. Dev.	95% conf. int.
β2m	3	704	2.516	2.618	1.14	2.534 - 2.702
Mef2b	3	780	2.444	2.546	1.05	2.472 - 2.620
Bcl6	3	620	2.565	2.629	1.09	2.544 - 2.715
Cd83	3	374	2.411	2.406	0.997	2.304 - 2.507
c-Myc	3	304	2.75	2.842	1.14	2.713 - 2.971
Pim1	3	750	2.335	2.407	0.992	2.336 - 2.478
Igλ	3	380	2.599	2.668	1.07	2.561 - 2.776

Supporting data for graphs in Figure 1C and 1D. For each gene analyzed relative to *Igh* the number of slides analyzed (Slides), number of measurements taken (Number), median distance (Median), mean distance (Mean), standard deviation (St. Dev.), and 95% confidence interval (95% conf. int.) are shown. Individual slides came from independent experiments. Only cells in which both alleles of both loci of interest were clearly labeled by FISH probes were included in analysis. Two measurements were taken from each cell.
