# Supplementary material for: AID-Targeting and Hypermutation of Non-Immunoglobulin Genes Does Not Correlate with Proximity to Immunoglobulin Genes in Germinal Center B Cells
Source: PLoS One. 2012 Jun 29;7(6):e39601. doi: 10.1371/journal.pone.0039601 (PMC3387148; doi:10.1371/journal.pone.0039601)
Supplement: Table S3 — KS tests of FISH data for distance to Igh in GC cells. Kolmogorov-Smirnov (KS) test results derived from the datasets of Figure 1C and 1E. KS tests, which compare dataset distributions, were done using the website http://www.physics.csbsju.edu/stats/KS-test.html. Datasets on the left were compared with datasets on the top. The p value is shown, and for any comparison that led to a p value less than 0.1, the corresponding D value is shown in parentheses. D is the maximum vertical distance between the two cumulative fraction plots and indicates the magnitude of the difference between the two distributions. (PDF) [file pone.0039601.s008.pdf]

Table S3. KS tests of FISH data for distance to *Igh* in GC cells.

	Bcl6	*Cd83*	*c-Myc*	*Pim1*	*Igλ*	*β2m*
β2m	0.7500	0.0610 (0.0839)	0.0230 (0.1015)	0.0090 (0.0854)	0.2040	-
Mef2b	0.3460	0.2830	0.0030 (0.1201)	0.1010	0.1130	0.4560

Kolmogorov-Smirnov (KS) test results derived from the datasets of Figure 1C and 1E. KS tests, which compare dataset distributions, were done using the website http://www.physics.csbsju.edu/stats/KS-test.html. Datasets on the left were compared with datasets on the top. The p value is shown, and for any comparison that led to a p value less than 0.1, the corresponding D value is shown in parentheses. D is the maximum vertical distance between the two cumulative fraction plots and indicates the magnitude of the difference between the two distributions.
