# Supplementary material for: AID-Targeting and Hypermutation of Non-Immunoglobulin Genes Does Not Correlate with Proximity to Immunoglobulin Genes in Germinal Center B Cells
Source: PLoS One. 2012 Jun 29;7(6):e39601. doi: 10.1371/journal.pone.0039601 (PMC3387148; doi:10.1371/journal.pone.0039601)
Supplement: Table S4 — Summary of FISH data for genes relative to Igh in naïve B cells. Supporting data for graphs in Figure 1E and 1F. See the legend of Table S2 for a full description. (PDF) [file pone.0039601.s009.pdf]

Table S4. Summary of FISH data for genes relative to *Igh* in naïve B cells.

	Slides	Number	Median	Mean	St. Dev.	95% conf. int.
β2m	2	278	2.122	2.144	0.777	2.052 - 2.235
Mef2b	3	416	1.839	1.848	0.740	1.776 - 1.919
Bcl6	2	224	2.029	2.033	0.717	1.939 - 2.128
Cd83	2	186	1.843	1.802	0.681	1.704 - 1.901
c-Myc	3	210	1.934	1.997	0.797	1.889 - 2.106
Pim1	3	434	1.704	1.736	0.751	1.665 - 1.807
Igλ	3	220	1.778	1.852	0.861	1.738 - 1.967

Supporting data for graphs in Figure 1E and 1F. See the legend of Table S2 for a full description.
