# Supplementary material for: AID-Targeting and Hypermutation of Non-Immunoglobulin Genes Does Not Correlate with Proximity to Immunoglobulin Genes in Germinal Center B Cells
Source: PLoS One. 2012 Jun 29;7(6):e39601. doi: 10.1371/journal.pone.0039601 (PMC3387148; doi:10.1371/journal.pone.0039601)
Supplement: Table S5 — KS tests of FISH data for genes relative to Igh in naïve cells. KS test results comparing the datasets in Figure 1E and 1F. See the legend of Table S3 for a full description. (PDF) [file pone.0039601.s010.pdf]

Table S5. KS tests of FISH data for genes relative to *Igh* in naïve cells.

	Bcl6	*Cd83*	*c-Myc*	*Pim1*	*Igλ*	*β2m*
β2m	0.2340	<0.00005 (0.1935)	0.0870 (0.1131)	<0.00005 (0.2209)	0.0020 (0.1635)	-
Mef2b	0.0010 (0.1566)	0.9620	0.2730	0.0420 (0.0947)	0.5140	<0.00005 (0.1647)

KS test results comparing the datasets in Figure 1E and 1F. See the legend of Table S3 for a full description.
