# Supplementary material for: AID-Targeting and Hypermutation of Non-Immunoglobulin Genes Does Not Correlate with Proximity to Immunoglobulin Genes in Germinal Center B Cells
Source: PLoS One. 2012 Jun 29;7(6):e39601. doi: 10.1371/journal.pone.0039601 (PMC3387148; doi:10.1371/journal.pone.0039601)
Supplement: Table S6 — Summary of FISH data for c-Myc relative to Igh at various timepoints. Supporting data for graphs in Figure 2A and 2B. See the legend of Table S2 for a full description. (PDF) [file pone.0039601.s011.pdf]

Table S6. Summary of FISH data for *c-Myc* relative to *Igh* at various timepoints.

	Slides	Number	Median	Mean	St. Dev.	95% conf. int.
day 8	1	102	3.164	3.14	1.24	2.896 - 3.384
day 10	1	100	2.775	2.863	1.14	2.637 - 3.089
day 12	1	98	2.669	2.771	1.21	2.529 - 3.013
day 14	1	108	2.693	2.857	1.12	2.643 - 3.070
day 16	1	110	3.087	3.124	1.1	2.916 - 3.333

Supporting data for graphs in Figure 2A and 2B. See the legend of Table S2 for a full description.
