# Supplementary material for: AID-Targeting and Hypermutation of Non-Immunoglobulin Genes Does Not Correlate with Proximity to Immunoglobulin Genes in Germinal Center B Cells
Source: PLoS One. 2012 Jun 29;7(6):e39601. doi: 10.1371/journal.pone.0039601 (PMC3387148; doi:10.1371/journal.pone.0039601)
Supplement: Table S7 — KS tests of FISH data for c-Myc relative to Igh at various timepoints. KS test results comparing the datasets in Figure 2A and 2B. See the legend of Table S3 for a full description. (PDF) [file pone.0039601.s012.pdf]

Table S7. KS tests of FISH data for *c-Myc* relative to *Igh* at various timepoints.

	day 10	day 12	day 14	day 16
day 8	0.1000	0.0050 (0.2385)	0.0990 (0.1661)	0.8660
day 10	-	0.5500	0.9540	0.2060
day 12	-	-	0.9010	0.0090 (0.2237)
day 14	-	-	-	0.0690

KS test results comparing the datasets in Figure 2A and 2B. See the legend of Table S3 for a full description.
