# Supplementary material for: AID-Targeting and Hypermutation of Non-Immunoglobulin Genes Does Not Correlate with Proximity to Immunoglobulin Genes in Germinal Center B Cells
Source: PLoS One. 2012 Jun 29;7(6):e39601. doi: 10.1371/journal.pone.0039601 (PMC3387148; doi:10.1371/journal.pone.0039601)
Supplement: Table S8 — Summary of FISH data for genes relative to Igλ in GC cells. Supporting data for graphs in Figure 3A and 3B. See the legend of Table S2 for a full description. (PDF) [file pone.0039601.s013.pdf]

Table S8. Summary of FISH data for genes relative to *Igλ* in GC cells.

	Slides	Number	Median	Mean	St. Dev.	95% conf. int.
β2m	3	194	2.763	2.813	1.09	2.658 - 2.968
Mef2b	3	294	2.180	2.172	0.868	2.072 - 2.272
Cd83	3	340	2.102	2.258	0.975	2.154 - 2.362
c-Myc	3	378	2.603	2.700	1.13	2.586 - 2.814
Pim1	2	322	2.064	2.203	1.03	2.091 - 2.316
Igh	3	380	2.605	2.674	1.04	2.569 - 2.779

Supporting data for graphs in Figure 3A and 3B. See the legend of Table S2 for a full description.
