# Supplementary material for: AID-Targeting and Hypermutation of Non-Immunoglobulin Genes Does Not Correlate with Proximity to Immunoglobulin Genes in Germinal Center B Cells
Source: PLoS One. 2012 Jun 29;7(6):e39601. doi: 10.1371/journal.pone.0039601 (PMC3387148; doi:10.1371/journal.pone.0039601)
Supplement: Table S9 — KS tests of FISH data for genes relative to Igλ in GC cells. KS test results comparing the datasets used in Figure 3A and 3B. See the legend of Table S3 for a full description. (PDF) [file pone.0039601.s014.pdf]

Table S9. KS tests of FISH data for genes relative to *Igλ* in GC cells.

	Cd83	*c-Myc*	*Pim1*	*Igh*	*β2m*
β2m	<0.00005 (0.2434)	0.5080	<0.00005 (0.2284)	0.4080	-
Mef2b	0.3510	<0.00005 (0.2060)	0.4880	<0.00005 (0.1989)	<0.00005 (0.2467)

KS test results comparing the datasets used in Figure 3A and 3B. See the legend of Table S3 for a full description.
