# Supplementary material for: AID-Targeting and Hypermutation of Non-Immunoglobulin Genes Does Not Correlate with Proximity to Immunoglobulin Genes in Germinal Center B Cells
Source: PLoS One. 2012 Jun 29;7(6):e39601. doi: 10.1371/journal.pone.0039601 (PMC3387148; doi:10.1371/journal.pone.0039601)
Supplement: Table S10 — Summary of FISH data for genes relative to Igλ in naïve cells. Number of slides analyzed, number of distances measured, and statistical analysis for each of the datasets included in Figure 3C and 3D. See the legend of Table S2 for a full description. (PDF) [file pone.0039601.s015.pdf]

Table S10. Summary of FISH data for genes relative to *Ig*λ in naïve cells.

	Slides	Number	Median	Mean	St. Dev.	95% conf. int.
β2m	3	504	2.181	2.213	0.870	2.137 - 2.289
Mef2b	2	432	1.825	1.874	0.806	1.797 - 1.950
Cd83	2	320	1.880	1.942	0.788	1.855 - 2.029
c-Myc	3	290	1.891	2.017	0.911	1.912 - 2.123
Pim1	3	786	1.898	1.966	0.869	1.905 - 2.026
Igh	3	220	1.767	1.805	0.809	1.697 - 1.912

Number of slides analyzed, number of distances measured, and statistical analysis for each of the datasets included in Figure 3C and 3D. See the legend of Table S2 for a full description.
