# Supplementary material for: AID-Targeting and Hypermutation of Non-Immunoglobulin Genes Does Not Correlate with Proximity to Immunoglobulin Genes in Germinal Center B Cells
Source: PLoS One. 2012 Jun 29;7(6):e39601. doi: 10.1371/journal.pone.0039601 (PMC3387148; doi:10.1371/journal.pone.0039601)
Supplement: Table S11 — KS tests of FISH data for genes relative to Igλ in naïve cells. KS test results comparing the datasets used in Figure 3C and 3D. See the legend of Table S3 for a full description. (PDF) [file pone.0039601.s016.pdf]

Table S11. KS tests of FISH data for genes relative to *Igλ* in naïve cells.

	Cd83	*c-Myc*	*Pim1*	*Igh*	*β2m*
β2m	<0.00005 (0.1527)	0.0010 (0.1395)	<0.00005 (0.1431)	<0.00005 (0.2067)	-
Mef2b	0.3840	0.28	0.1330	0.8430	<0.00005 (0.1819)

KS test results comparing the datasets used in Figure 3C and 3D. See the legend of Table S3 for a full description.
