# Supplementary material for: AID-Targeting and Hypermutation of Non-Immunoglobulin Genes Does Not Correlate with Proximity to Immunoglobulin Genes in Germinal Center B Cells
Source: PLoS One. 2012 Jun 29;7(6):e39601. doi: 10.1371/journal.pone.0039601 (PMC3387148; doi:10.1371/journal.pone.0039601)
Supplement: Table S12 — Summary of FISH data for Myc31 relative to other genes. Supporting data for graph in Figure S4A. See the legend of Table S2 for a full description. (PDF) [file pone.0039601.s017.pdf]

Table S12. Summary of FISH data for Myc31 relative to other genes.

Gene	Population	Slides	Number	Median	Mean	Std. Dev.	95% conf. int.
c-Myc	GC	1	35	2.42	2.473	1.058	2.109-2.836
c-Myc	Naive	2	127	2.367	2.321	0.8549	2.171-2.471
Igh	GC	2	58	2.618	2.679	1.102	2.390-2.969
Igh	Naive	2	67	2.298	2.197	0.7769	2.007-2.386
Igλ	GC	2	167	2.638	2.619	1.021	2.463-2.775
Igλ	Naive	2	135	2.22	2.268	0.9042	2.114-2.422
Igκ	GC	2	105	2.545	2.752	1.191	2.522-2.983
Igκ	Naive	2	128	2.255	2.281	0.8218	2.137-2.425

Supporting data for graph in Figure S4A. See the legend of Table S2 for a full description.
