# Supplementary material for: AID-Targeting and Hypermutation of Non-Immunoglobulin Genes Does Not Correlate with Proximity to Immunoglobulin Genes in Germinal Center B Cells
Source: PLoS One. 2012 Jun 29;7(6):e39601. doi: 10.1371/journal.pone.0039601 (PMC3387148; doi:10.1371/journal.pone.0039601)
Supplement: Table S13 — Summary of FISH data for Myc24 relative to other genes. Supporting data for graph in Figure S4B. See the legend of Table S2 for a full description. (PDF) [file pone.0039601.s018.pdf]

Table S13. Summary of FISH data for Myc24 relative to other genes.

Gene	Population	Slides	Number	Median	Mean	Std. Dev.	95% Conf. Int.
c-Myc	GC	1	87	2.665	2.682	1.033	2.462-2.903
c-Myc	naive	1	67	2.692	2.622	0.8265	2.421-2.824
Igh	GC	2	59	2.726	2.95	1.129	2.655-3.244
Igh	naive	2	196	2.85	2.931	1.021	2.787-3.074
Igλ	GC	2	111	3.027	3.004	1.092	2.799-3.21
Igλ	naive	2	217	2.846	2.812	0.9683	2.683-2.942
Igκ	GC	2	113	3.111	3.053	1.244	2.821-3.285
Igκ	naive	2	87	2.765	2.716	0.9759	2.508-2.924

Supporting data for graph in Figure S4B. See the legend of Table S2 for a full description.
