# Supplementary material for: AID-Targeting and Hypermutation of Non-Immunoglobulin Genes Does Not Correlate with Proximity to Immunoglobulin Genes in Germinal Center B Cells
Source: PLoS One. 2012 Jun 29;7(6):e39601. doi: 10.1371/journal.pone.0039601 (PMC3387148; doi:10.1371/journal.pone.0039601)
Supplement: Table S14 — Mutation in B1-8 het Myc31+ splenic GC cells. Supporting data for left side of graph in Figure 5A. See the legend of Table S1 for a full description. (PDF) [file pone.0039601.s019.pdf]

Table S14. Mutation in B1-8 het Myc31$^+$ splenic GC cells.

Gene	Sample	Mut	bp	Frequency	p<0.05
β2m	2	2	40180	-	-
β2m	3	1	38062	-	-
β2m	total	3	78242	3.83 E-05	No
Bcl6	2	4	37168	-	-
Bcl6	3	12	37095	-	-
Bcl6	total	16	74263	21.5 E-05	Yes
Mouse *c-Myc*	1	5	15633	-	-
Mouse *c-Myc*	2	2	43262	-	-
Mouse *c-Myc*	3	3	40909	-	-
Mouse *c-Myc*	total	10	99804	10.0 E -05	Yes
huMyc31	2	3	35925	-	-
huMyc31	3	0	38463	-	-
huMyc31	total	3	74388	4.03 E-05	No
VJλ1	1	61	16084	-	-
VJλ1	2	71	16634	-	-
VJλ1	3	69	16614	-	-
VJλ1	total	201	49332	407 E-05	Yes

Supporting data for left side of graph in Figure 5A. See the legend of Table S1 for a full description.
