# Supplementary material for: AID-Targeting and Hypermutation of Non-Immunoglobulin Genes Does Not Correlate with Proximity to Immunoglobulin Genes in Germinal Center B Cells
Source: PLoS One. 2012 Jun 29;7(6):e39601. doi: 10.1371/journal.pone.0039601 (PMC3387148; doi:10.1371/journal.pone.0039601)
Supplement: Table S15 — Mutation in B1-8 het Myc24+ splenic GC cells. Supporting data for right half of graph in Figure 5A. See the legend of Table S1 for a full description. (PDF) [file pone.0039601.s020.pdf]

Table S15. Mutation in B1-8 het Myc24$^+$ splenic GC cells.

Gene	Sample	Mut	bp	Frequency	$p<0.05$
β2m	1	1	38011	-	
β2m	2	0	32945	-	
β2m	total	1	70956	1.41 E-05	No
Bcl6	1	7	35766	-	
Bcl6	2	2	29896	-	
Bcl6	total	9	65662	13.7 E-05	Yes
mouse *c-Myc*	1	1	39828	-	
mouse *c-Myc*	2	0	20082	-	
mouse *c-Myc*	total	1	59910	1.67 E-05	No
huMyc24	1	2	86457	-	
huMyc24	2	1	19301	-	
huMyc24	total	3	105758	2.84 E-05	No
VJλ1	1	47	13579	-	
VJλ1	2	59	18792	-	
VJλ1	total	106	32371	327 E-05	Yes

Supporting data for right half of graph in Figure 5A. See the legend of Table S1 for a full description.
