# Supplementary material for: AID-Targeting and Hypermutation of Non-Immunoglobulin Genes Does Not Correlate with Proximity to Immunoglobulin Genes in Germinal Center B Cells
Source: PLoS One. 2012 Jun 29;7(6):e39601. doi: 10.1371/journal.pone.0039601 (PMC3387148; doi:10.1371/journal.pone.0039601)
Supplement: Table S16 — Mutation in Myc31+ Peyer's patch GC B cells. Supporting data for red bars in the left half of the graph in Figure 5B. See the legend of Table S1 for a full description. (PDF) [file pone.0039601.s021.pdf]

Table S16. Mutation in Myc31^{+} Peyer's patch GC B cells.

Gene	Sample	Mut	Bp	Frequency	p<0.05
β2m	2	0	21075	0	No
Bcl6	1	8	39187	20.4 E-05	Yes
Mouse *c-Myc*	1	6	22803	-	-
Mouse *c-Myc*	2	4	58035	-	-
Mouse *c-Myc*	3	4	56048	-	-
Mouse *c-Myc*	total	14	136886	10.2 E-05	Yes
huMyc31	1	3	87412	-	-
huMyc31	2	0	13978	-	-
huMyc31	3	0	42657	-	-
huMyc31	total	3	144047	2.08 E-05	No
Igh Jh4 intron	1	93	12562	-	-
Igh Jh4 intron	3	201	15922	-	-
Igh Jh4 intron	total	294	28484	1032 E-05	Yes

Supporting data for red bars in the left half of the graph in Figure 5B. See the legend of Table S1 for a full description.
