# Supplementary material for: AID-Targeting and Hypermutation of Non-Immunoglobulin Genes Does Not Correlate with Proximity to Immunoglobulin Genes in Germinal Center B Cells
Source: PLoS One. 2012 Jun 29;7(6):e39601. doi: 10.1371/journal.pone.0039601 (PMC3387148; doi:10.1371/journal.pone.0039601)
Supplement: Table S17 — Mutation in Myc24+ Peyer's patch GC B cells. Supporting data for red bars in the right half of the graph in Figure 5B. See the legend of Table S1 for a full description. (PDF) [file pone.0039601.s022.pdf]

Table S17. Mutation in Myc24^{+} Peyer's patch GC B cells.

Gene	Sample	Mut	bp	Frequency	p<0.05
β2m	3	0	18317	0	No
Bcl6	1	10	45576	21.9 E-05	Yes
Mouse *c-Myc*	1	0	18277	-	-
Mouse *c-Myc*	2	2	21785	-	-
Mouse *c-Myc*	3	4	40997	-	-
Mouse *c-Myc*	total	6	81059	7.40 E-05	Yes
huMyc24	1	0	88880	-	-
huMyc24	2	2	94237	-	-
huMyc24	3	0	42267	-	-
huMyc24	total	2	225384	0.887 E-05	No
Igh Jh4 intron	1	48	11167	-	-
Igh Jh4 intron	2	157	13840	-	-
Igh Jh4 intron	3	90	11166	-	-
Igh Jh4 intron	total	295	36173	815 E-05	Yes

Supporting data for red bars in the right half of the graph in Figure 5B. See the legend of Table S1 for a full description.
