# Supplementary material for: AID-Targeting and Hypermutation of Non-Immunoglobulin Genes Does Not Correlate with Proximity to Immunoglobulin Genes in Germinal Center B Cells
Source: PLoS One. 2012 Jun 29;7(6):e39601. doi: 10.1371/journal.pone.0039601 (PMC3387148; doi:10.1371/journal.pone.0039601)
Supplement: Table S18 — Mutation in Myc31+Msh2−/−Ung−/− Peyer's patch GC B cells. Supporting data for yellow bars in the left half of Figure 5B. See the legend of Table S1 for a full description. (PDF) [file pone.0039601.s023.pdf]

**Table S18. Mutation in Myc31$^{+}$Msh2$^{-/-}$Ung$^{-/-}$ Peyer's patch GC B cells.**

| Gene | Sample | Mut | bp | Frequency | p<0.05 |
|---|---|---|---|---|---|
| *β2m* | 1 | 1 | 21029 | - | - |
| *β2m* | 3 | 1 | 38094 | - | - |
| *β2m* | 4 | 1 | 36389 | - | - |
| *β2m* | total | 3 | 95512 | 3.14 E-05 | No |
| *Bcl6* | 1 | 112 | 89640 | - | - |
| *Bcl6* | 3 | 48 | 37683 | - | - |
| *Bcl6* | 4 | 38 | 40180 | - | - |
| *Bcl6* | total | 198 | 167503 | 118 E-05 | Yes |
| mouse *c-Myc* | 1 | 73 | 90289 | - | - |
| mouse *c-Myc* | 2 | 36 | 45650 | - | - |
| mouse *c-Myc* | 3 | 29 | 69278 | - | - |
| mouse *c-Myc* | 4 | 17 | 23042 | - | - |
| mouse *c-Myc* | total | 155 | 228259 | 67.9 E-05 | Yes |
| huMyc31 | 1 | 1 | 92998 | - | - |
| huMyc31 | 2 | 0 | 30439 | - | - |
| huMyc31 | 3 | 1 | 25627 | - | - |
| huMyc31 | 4 | 0 | 24175 | - | - |
| huMyc31 | total | 2 | 173239 | 1.15 E-05 | No |
| huMyc31 intron | 1 | 0 | 47992 | - | - |
| huMyc31 intron | 2 | 2 | 47920 | - | - |
| huMyc31 intron | 3 | 0 | 48059 | - | - |
| huMyc31 intron | 4 | 1 | 48110 | - | - |
| huMyc31 intron | total | 3 | 192081 | 1.56 E-05 | No |
| *Igh* Jh4 intron | 1 | 84 | 24528 | - | - |
| *Igh* Jh4 intron | 2 | 58 | 15026 | - | - |
| *Igh* Jh4 intron | 3 | 49 | 14736 | - | - |
| *Igh* Jh4 intron | 4 | 32 | 14468 | - | - |
| *Igh* Jh4 intron | total | 223 | 68758 | 324 E-05 | Yes |

Supporting data for yellow bars in the left half of Figure 5B. See the legend of Table S1 for a full description.
