# Supplementary material for: AID-Targeting and Hypermutation of Non-Immunoglobulin Genes Does Not Correlate with Proximity to Immunoglobulin Genes in Germinal Center B Cells
Source: PLoS One. 2012 Jun 29;7(6):e39601. doi: 10.1371/journal.pone.0039601 (PMC3387148; doi:10.1371/journal.pone.0039601)
Supplement: Table S19 — Mutation in Myc24+Msh2−/−Ung−/− Peyer's patch GC B cells. Supporting data for yellow bars in the right half of the graph in Figure 5B. See the legend of Table S1 for a full description. (PDF) [file pone.0039601.s024.pdf]

Table S19. Mutation in Myc24^{+}Msh2$^{-/-}$Ung$^{-/-}$ Peyer's patch GC B cells.

Gene	Sample	Mut	bp	Frequency	p<0.05
β2m	1	0	38557	-	-
β2m	2	2	37916	-	-
β2m	3	1	14894	-	-
β2m	total	3	91367	3.28 E-05	No
Bcl6	1	65	57218	114 E-05	Yes
mouse *c-Myc*	1	48	89111	-	-
mouse *c-Myc*	2	33	57081	-	-
mouse *c-Myc*	3	41	56355	-	-
mouse *c-Myc*	total	122	202547	60.2 E-05	Yes
huMyc24	1	1	93238	-	-
huMyc24	2	0	19922	-	-
huMyc24	3	0	22945	-	-
huMyc24	total	1	136105	0.735 E-05	No
Igh Jh4 intron	1	86	25563	-	-
Igh Jh4 intron	2	36	6279	-	-
Igh Jh4 intron	3	39	5235	-	-
Igh Jh4 intron	total	161	37077	434 E-05	Yes

Supporting data for yellow bars in the right half of the graph in Figure 5B. See the legend of Table S1 for a full description.
