# Supplementary material for: AID-Targeting and Hypermutation of Non-Immunoglobulin Genes Does Not Correlate with Proximity to Immunoglobulin Genes in Germinal Center B Cells
Source: PLoS One. 2012 Jun 29;7(6):e39601. doi: 10.1371/journal.pone.0039601 (PMC3387148; doi:10.1371/journal.pone.0039601)
Supplement: Table S20 — Summary of FISH data for c-Myc relative to Igκ. Supporting data for Igκ data in Figure S5. See the legend of Table S2 for a full description. (PDF) [file pone.0039601.s025.pdf]

Table S20. Summary of FISH data for *c-Myc* relative to *Igκ*.

	Slides	Number	Median	Mean	St. Dev.	95% conf. int.
GC	3	416	2.737	2.749	1.05	2.648-2.850
Naive	3	442	2.292	2.383	0.951	2.294-2.471

Supporting data for *Igκ* data in Figure S5. See the legend of Table S2 for a full description.
