# Supplementary material for: AID-Targeting and Hypermutation of Non-Immunoglobulin Genes Does Not Correlate with Proximity to Immunoglobulin Genes in Germinal Center B Cells
Source: PLoS One. 2012 Jun 29;7(6):e39601. doi: 10.1371/journal.pone.0039601 (PMC3387148; doi:10.1371/journal.pone.0039601)
Supplement: Table S21 — KS tests of FISH data for c-Myc relative to Ig loci in GC B cells. KS test results comparing the GC cell datasets in Figure S5. See the legend of Table S3 for a full description. (PDF) [file pone.0039601.s026.pdf]

Table S21. KS tests of FISH data for *c-Myc* relative to Ig loci in GC B cells.

	Igλ	*Igh*
Igκ	0.376	0.207
Igλ	-	0.279

KS test results comparing the GC cell datasets in Figure S5. See the legend of Table S3 for a full description.
