# Supplementary material for: AID-Targeting and Hypermutation of Non-Immunoglobulin Genes Does Not Correlate with Proximity to Immunoglobulin Genes in Germinal Center B Cells
Source: PLoS One. 2012 Jun 29;7(6):e39601. doi: 10.1371/journal.pone.0039601 (PMC3387148; doi:10.1371/journal.pone.0039601)
Supplement: Table S22 — KS tests of FISH data for c-Myc relative to Ig loci in naive B cells. KS test results comparing the naïve cell datasets in Figure S5. See the legend of Table S3 for a full description. (PDF) [file pone.0039601.s027.pdf]

**Table S22. KS tests of FISH data for *c-Myc* relative to Ig loci in naive B cells.**

| | *Igλ* | *Igh* |
|---|---|---|
| *Igκ* | <0.00005 (0.1863) | <0.00005 (0.2242) |
| *Igλ* | - | 0.4900 |

KS test results comparing the naïve cell datasets in Figure S5. See the legend of Table S3 for a full description.
