# Supplementary material for: AID-Targeting and Hypermutation of Non-Immunoglobulin Genes Does Not Correlate with Proximity to Immunoglobulin Genes in Germinal Center B Cells
Source: PLoS One. 2012 Jun 29;7(6):e39601. doi: 10.1371/journal.pone.0039601 (PMC3387148; doi:10.1371/journal.pone.0039601)
Supplement: Table S23 — BACs used for FISH analysis. BACs used for FISH analysis are listed (BAC Name) along with their accession numbers (Accession), size in kb (Size), and position (Relative position) with reference to the gene of interest (Gene). The chromosome number on which each gene is found (Chr) is listed, as are the mutation groups of each gene as determined by Liu et al. [14]. (PDF) [file pone.0039601.s028.pdf]

Table S23. BACs used for FISH analysis.

Gene	Chr	Group	BAC Name	Accession	Size	Relative position
Bcl6	16	I, A	RP23-274H19	AC158397.2	198	contains *Bcl6*
Bcl6	16	I, A	RP23-137L8	AC169509.1	206	76 kb downstream
Cd83	13	I, A	RP23-349P1	AC091785.5	265	overlaps *Cd83*
Cd83	13	I, A	RP24-156M16	AC124465.6	170	21 kb upstream
c-Myc	15	I, A	RP23-457I7	AC153008.4	172	contains *c-Myc*
c-Myc	15	I, A	RP23-342F3	AC126552.5	214	224 kb downstream
Pim1	17	I, A	RP23-73B23	AC162182.2	317	7 kb upstream
Pim1	17	I, A	RP24-271E12	AC154279.2	174	44 kb downstream
β*2m*	2	III, C	RP23-277B16	AL844573.4	194	contains β*2m*
β*2m*	2	III, C	RP23-34E24	AL845457.16	232	contains β*2m*
Mef2b	8	III, C	RP24-490I13	AC124327.9	174	contains *Mef2b*
Mef2b	8	III, C	RP23-228G4	-	232	143 kb downstream
Igh	12	-	BAC199	-	-	3' end of Igh
Igκ	6	-	RP24-387E13	-	138	contains *C*κ, *J*κ
Igλ	16	-	RP23-357J7	AC113264.21	223	upstream of *Ig*λ
Igλ	16	-	RP23-247I11	AC079817.41	197	contains *C*λ*1*, *V*λ*1*
huMyc	-	-	CTD-3056O22	AC103819.3	148	contains *c-Myc*

BACs used for FISH analysis are listed (BAC Name) along with their accession numbers (Accession), size in kb (Size), and position (Relative position) with reference to the gene of interest (Gene). The chromosome number on which each gene is found (Chr) is listed, as are the mutation groups of each gene as determined by Liu et al. [14].
